# Supplementary material for: COVID-19 Global Risk: Expectation vs. Reality
Source: Int J Environ Res Public Health. 2020 Aug 3;17(15):5592. doi: 10.3390/ijerph17155592 (PMC7432363; doi:10.3390/ijerph17155592)
Supplement: Supplementary file 1 [file ijerph-17-05592-s001.zip › ijerph-844790-supplementary.html]

COVID-19 Global Risk: Expectation vs Reality


# COVID-19 Global Risk: Expectation vs Reality

#### Mudassar Arsalan

#### July 13, 2020

- 1 Library Initilization
- 2 Base Data Loading from the Local Drive
  - 2.1  Base Data Table
- 3 Covid -19 Regression Model Function
- 4 Pre-COVID19 - Pairwise Risk Assessment
  - 4.1  Pairwise Risk Evaluation Function
  - 4.2  Pre-COVID19 Weights Extraction
  - 4.3  Pre-COVID-19 Risk Evaluation
  - 4.4  Pre-COVID-19 Risk Results
- 5 Post-COVID-19 - Regression Analysis
  - 5.1  Post-COVID-19 Risk Evaluation Function
  - 5.2  Post-COVID-19 Static Model 
    - 5.2.1  Static Weights Extraction
    - 5.2.2  Static Risk Evaluation
    - 5.2.3  Static Risk Results
  - 5.3  Post-COVID-19 Dynamic Model 
    - 5.3.1  Dynamic Weights Extraction
    - 5.3.2  Dynamic Risk Evaluation
    - 5.3.3  Dynamic Risk Results
- 6 Difference of Pre COVID and Post COVID Dynamic Risk
- 7 List of Post-COVID-19 Top Countries with Level of Risk

---

# 1 Library Initilization

```
setwd("D:/VS/COVIDModel")
.libPaths("c:/R/win-library/3.6")
library(MASS)
library(EnvStats)
library(stringr)
library(bestNormalize)
library(Ecfun)
#library(COVID19)
library(covid19.analytics)
library(jsonlite)
library(zoo)
library(tidyverse)
library(stringr)
library(PerformanceAnalytics)
library(anytime)
library(caret)
library(relaimpo)
library(stargazer)
library(car)
library(kableExtra)
library(MCDA)
library(ahp)
library(sp)
library(sf)
source('./RSCript/COVIDFunctions.R')
```

# 2 Base Data Loading from the Local Drive

```
setwd("D:/VS/COVIDModel")
#Stringency Index Data
SI.date = "2020-05-13"
si_source <- read.csv("D:/VS/COVIDModel/Data/si_data_rev_14may.csv", stringsAsFactors = FALSE, check.names = FALSE, fileEncoding = "UTF-8-BOM")

# Country Codes
CountryCodeList <- read.csv("d:/VS/COVIDModel/data/country_JHU.csv", stringsAsFactors = FALSE, check.names = FALSE, fileEncoding = "UTF-8-BOM")


#World Bank Indicators
BaseData <- read.csv("D:/VS/COVIDModel/data/BaseData.csv", stringsAsFactors = FALSE, check.names = FALSE, fileEncoding = "UTF-8-BOM")
BaseData <- BaseData[, c(1:2, 5:12)]

#Human Development Index Data
HDI <- read.csv("D:/VS/COVIDModel/data/HDI_country.csv", stringsAsFactors = FALSE, check.names = FALSE, fileEncoding = "UTF-8-BOM")
HDI_data <- HDI[, c("country_code", "2018")]
colnames(HDI_data) <- c("country_code", "HDI")
BaseData <- merge(BaseData, HDI_data, by = "country_code", all.x = TRUE)

#Human Capital Index
HCI <- read.csv("D:/VS/COVIDModel/data/HCI_country.csv", stringsAsFactors = FALSE, check.names = FALSE, fileEncoding = "UTF-8-BOM")
HCI_data <- HCI[, c("country_code", "HCI")]
BaseData <- merge(BaseData, HCI_data, by = "country_code", all.x = TRUE)

# Disability-Adjusted Life Year
DALYs <- read.csv("D:/VS/COVIDModel/data/DALYs.csv", stringsAsFactors = FALSE, check.names = FALSE, fileEncoding = "UTF-8-BOM")
GHDx_Countries <- read.csv("D:/VS/COVIDModel/data/GHDx_Countries_Regions.csv", stringsAsFactors = FALSE, check.names = FALSE, fileEncoding = "UTF-8-BOM")
DALYs <- merge(DALYs, GHDx_Countries, by = "location_id", all.x = TRUE)
DALYs <- DALYs[, c("country_code", "val")]
colnames(DALYs) <- c("country_code", "DALYs")
BaseData <- merge(BaseData, DALYs, by = "country_code", all.x = TRUE)

si <- si_source[c("country_code", SI.date)]
colnames(si) <- c("country_code", "stringency_wt")
BaseData <- merge(BaseData, si, by = "country_code", all.x = TRUE)
BaseData_ss = BaseData[, c(1, 3:13)]
```

## 2.1 Base Data Table

```
kable(BaseData) %>%
     kable_styling("striped", full_width = F, fixed_thead = T) %>%
     row_spec(0) %>%
     scroll_box(width = "910px", height = "440px")
```

| country\_code | CNTRY\_NAME | Pop\_2018 | GDP\_PC | A65abp\_2018 | PopDen\_Avg | Physician | Nurses | HospitalBeds | Expenditure | HDI | HCI | DALYs | stringency\_wt |
| --- | --- | --- | --- | --- | --- | --- | --- | --- | --- | --- | --- | --- | --- |
| ABW | Aruba | 105845 | 25630.2665 | 13.550947 | 3689.0000 | 1.12000 | 3.12810 | 2.900000 | 29.647642 | NA | NA | NA | 69.99222 |
| AFG | Afghanistan | 37172386 | 520.8966 | 2.584927 | 7401.9054 | 0.28400 | 0.32000 | 0.500000 | 36.652642 | 0.496 | 0.389 | 49634.29 | 65.60630 |
| AGO | Angola | 30809762 | 3432.3857 | 2.216374 | 18571.4761 | 0.21490 | 1.31230 | 0.800000 | 16.671137 | 0.574 | 0.361 | 46502.58 | 26.76743 |
| ALB | Albania | 2866376 | 5268.8485 | 13.744736 | 6669.7894 | 1.19980 | 3.59980 | 2.900000 | 23.349881 | 0.791 | 0.621 | 27819.79 | 40.22388 |
| AND | Andorra | 77006 | 42029.7627 | 16.180000 | 2002.7870 | 3.33330 | 4.01280 | 2.500000 | 37.756894 | 0.857 | NA | 24265.59 | 66.77481 |
| ARE | United Arab Emirates | 9630959 | 43004.9534 | 1.085001 | 889.0000 | 2.39440 | 5.58570 | 1.200000 | 4.040008 | 0.866 | 0.659 | 22964.95 | 20.51222 |
| ARG | Argentina | 44494502 | 11683.9496 | 11.117789 | 3283.1201 | 3.96000 | 2.58000 | 5.000000 | 24.317775 | 0.830 | 0.611 | 26785.80 | 40.81880 |
| ARM | Armenia | 2951776 | 4212.0709 | 11.253818 | 3867.1812 | 2.89900 | 5.60940 | 4.200000 | 22.120846 | 0.760 | 0.572 | 30106.52 | NA |
| ASM | American Samoa | 55465 | 11466.6907 | 6.240000 | 1022.0000 | 0.78100 | 3.54840 | 2.600000 | 28.251402 | NA | NA | 28049.05 | NA |
| ATG | Antigua and Barbuda | 96286 | 16726.9808 | 8.799826 | 2500.0000 | 2.76470 | 3.11760 | 3.800000 | 29.647642 | 0.776 | NA | 25675.70 | NA |
| AUS | Australia | 24992369 | 57373.6867 | 15.656475 | 1693.5530 | 3.58740 | 12.66120 | 3.800000 | 26.117262 | 0.938 | 0.803 | 24508.29 | 34.89946 |
| AUT | Austria | 8847037 | 51461.9548 | 19.001566 | 3232.3376 | 5.14410 | 8.17780 | 7.600000 | 44.181771 | 0.914 | 0.793 | 27251.82 | 52.57904 |
| AZE | Azerbaijan | 9942334 | 4721.1781 | 6.195183 | 5296.2390 | 3.44660 | 6.96210 | 4.700000 | 27.958731 | 0.754 | 0.597 | 31553.63 | 23.21431 |
| BDI | Burundi | 11175378 | 271.7520 | 2.246940 | 9254.3940 | 0.05000 | 0.68000 | 0.800000 | 20.647628 | 0.423 | 0.380 | 50800.22 | 20.15967 |
| BEL | Belgium | 11422068 | 47518.6360 | 18.788744 | 2651.8007 | 3.32340 | 11.10110 | 6.200000 | 39.573699 | 0.919 | 0.757 | 28213.32 | 34.54512 |
| BEN | Benin | 11485048 | 901.5439 | 3.253605 | 3301.5703 | 0.15720 | 0.61450 | 0.500000 | 12.562992 | 0.520 | 0.406 | 48486.79 | 17.40293 |
| BFA | Burkina Faso | 19751535 | 715.1229 | 2.406981 | 5164.7966 | 0.06000 | 0.56960 | 0.400000 | 19.532167 | 0.434 | 0.369 | 61147.47 | 72.56256 |
| BGD | Bangladesh | 161356039 | 1698.2628 | 5.158391 | 21517.8127 | 0.52680 | 0.30670 | 0.800000 | 9.418916 | 0.614 | 0.479 | 29275.39 | 27.37832 |
| BGR | Bulgaria | 7024216 | 9272.6293 | 21.021914 | 1196.4233 | 3.98790 | 5.30290 | 6.800000 | 32.332434 | 0.816 | 0.676 | 41673.69 | 25.01360 |
| BHR | Bahrain | 1569439 | 24050.7575 | 2.426334 | 15024.8524 | 0.92570 | 2.49440 | 2.000000 | 20.176116 | 0.838 | 0.668 | 18543.73 | 30.66783 |
| BHS | Bahamas | 385640 | 32217.8716 | 7.257602 | 1312.1584 | 1.93730 | 3.13860 | 2.900000 | 17.936746 | 0.805 | NA | 29329.62 | NA |
| BIH | Bosnia and Herzegovina | 3323929 | 6065.6727 | 16.470317 | 2000.0000 | 2.00030 | 6.29980 | 3.500000 | 35.131803 | 0.769 | 0.618 | 34266.70 | 42.20474 |
| BLR | Belarus | 9485386 | 6289.9386 | 14.845148 | 3499.7009 | 4.07720 | 11.43830 | 11.000000 | 29.321605 | 0.817 | NA | 39002.72 | NA |
| BLZ | Belize | 383071 | 4884.7424 | 4.736459 | 2000.0000 | 1.12620 | 1.69470 | 1.300000 | 27.568219 | 0.720 | NA | 25909.81 | 32.94995 |
| BMU | Bermuda | 63968 | 85748.0000 | 18.530000 | 1246.0000 | 1.76600 | 9.22780 | 6.300000 | 19.846098 | NA | NA | 25512.08 | 39.84960 |
| BOL | Bolivia (Plurinational State of) | 11353142 | 3548.5901 | 7.191947 | 1646.0942 | 1.61110 | 0.73570 | 1.100000 | 21.811043 | 0.703 | NA | 28080.68 | 118.16786 |
| BRA | Brazil | 209469333 | 8920.7621 | 8.922838 | 5849.8937 | 2.14990 | 9.70830 | 2.200000 | 34.928812 | 0.761 | 0.560 | 28555.59 | 52.69659 |
| BRB | Barbados | 286641 | 17949.2815 | 15.802694 | 660.0000 | 2.48860 | 6.02730 | 5.800000 | 35.212924 | 0.813 | NA | 29578.63 | 38.19656 |
| BRN | Brunei Darussalam | 428962 | 31628.3288 | 4.873148 | 715.0000 | 1.77010 | 6.60120 | 2.700000 | 25.421083 | 0.845 | NA | 22350.00 | 36.44102 |
| BTN | Bhutan | 754394 | 3243.2311 | 6.003012 | 623.0000 | 0.37150 | 1.50940 | 1.700000 | 18.453961 | 0.617 | NA | 24255.56 | NA |
| BWA | Botswana | 2254126 | 8258.6417 | 4.223874 | 3000.0000 | 0.36880 | 3.30040 | 1.800000 | 23.638998 | 0.728 | 0.424 | 34146.00 | 27.97824 |
| CAF | Central African Republic | 4666377 | 475.7212 | 2.825774 | 6726.2260 | 0.06290 | 0.20390 | 1.000000 | 7.894358 | 0.381 | NA | 89691.78 | NA |
| CAN | Canada | 37058856 | 46232.9896 | 17.232007 | 2707.8907 | 2.61020 | 9.90560 | 2.700000 | 17.324548 | 0.922 | 0.799 | 25960.51 | 18.01809 |
| CHE | Switzerland | 8516543 | 82796.5472 | 18.623217 | 2879.0918 | 4.23630 | 17.28280 | 4.700000 | 16.971559 | 0.946 | 0.767 | 24062.56 | 55.14040 |
| CHL | Chile | 18729160 | 15923.3587 | 11.529802 | 3755.7118 | 1.08000 | 0.86000 | 2.200000 | 22.403473 | 0.847 | 0.674 | 24599.10 | 88.34206 |
| CHN | China | 1392730000 | 9770.8471 | 10.920884 | 9870.0050 | 1.78550 | 2.30730 | 4.200000 | 28.251402 | 0.758 | 0.673 | 26300.21 | 75.96876 |
| CIV | CÃ´te d’Ivoire | 25069229 | 1715.5313 | 2.858943 | 1858.3258 | 0.23260 | 0.85170 | 0.400000 | 20.575553 | 0.516 | 0.353 | 50386.54 | 71.09026 |
| CMR | Cameroon | 25216237 | 1533.7421 | 2.728877 | 16124.0000 | 0.08980 | 0.93370 | 1.300000 | 10.854785 | 0.563 | 0.394 | 44848.25 | 52.02868 |
| COD | Democratic Republic of the Congo | 84068091 | 561.7772 | 3.017841 | 7959.4679 | 0.09000 | 0.47000 | 0.800000 | 11.498314 | 0.459 | 0.369 | 54769.25 | 26.24274 |
| COG | Congo | 5244363 | 2147.7695 | 2.681720 | 3358.8594 | 0.11590 | 1.74310 | 1.600000 | 20.101405 | 0.609 | 0.420 | 45188.09 | 37.58853 |
| COL | Colombia | 49648685 | 6667.7907 | 8.478047 | 4636.3469 | 2.08350 | 1.26270 | 1.500000 | 25.257438 | 0.761 | 0.593 | 21692.63 | 31.57038 |
| COM | Comoros | 832322 | 1415.2636 | 3.007009 | 1190.0000 | 0.16990 | 0.92140 | 2.200000 | 20.101405 | 0.538 | 0.409 | 32888.07 | NA |
| CPV | Cabo Verde | 543767 | 3635.4068 | 4.609327 | 3000.0000 | 0.76940 | 1.22720 | 2.100000 | 28.424829 | 0.651 | NA | 25869.86 | 179.07766 |
| CRI | Costa Rica | 4999441 | 12027.3659 | 9.549848 | 4797.9887 | 1.14970 | 0.79570 | 1.200000 | 30.020615 | 0.794 | 0.619 | 21431.68 | 40.76266 |
| CUB | Cuba | 11338138 | 8821.8189 | 15.186211 | 2204.3281 | 8.19000 | 7.79000 | 5.200000 | 22.383506 | 0.778 | NA | 27872.50 | 37.48511 |
| CYM | Cayman Islands | 64174 | 81124.5135 | 13.130000 | 1278.0000 | 1.93900 | 3.12810 | 3.000000 | 29.647642 | NA | NA | NA | NA |
| CYP | Cyprus | 1189265 | 28159.3017 | 13.719062 | 1792.3269 | 1.95110 | 5.25170 | 3.400000 | 35.415707 | 0.873 | 0.751 | 23020.89 | 64.79993 |
| CZE | Czechia | 10625695 | 23078.5735 | 19.420877 | 2005.1308 | 4.31400 | 8.40710 | 6.500000 | 32.102007 | 0.891 | 0.782 | 32123.76 | 36.15922 |
| DEU | Germany | 82927922 | 47603.0276 | 21.461962 | 2037.3642 | 4.20870 | 13.19670 | 8.300000 | 28.170595 | 0.939 | 0.795 | 30834.29 | 31.28597 |
| DJI | Djibouti | 958920 | 3082.5431 | 4.527579 | 3439.2612 | 0.22030 | 0.53500 | 1.400000 | 22.383506 | 0.495 | NA | 33630.96 | 114.96951 |
| DMA | Dominica | 71625 | 7691.3451 | 11.430000 | 391.7141 | 1.08250 | 5.89990 | 3.800000 | 22.383506 | 0.724 | NA | 33886.40 | 76.75302 |
| DNK | Denmark | 5797446 | 61350.3479 | 19.812953 | 2733.8476 | 4.45670 | 10.30040 | 2.500000 | 37.586034 | 0.930 | 0.774 | 28111.42 | 62.95395 |
| DOM | Dominican Republic | 10627165 | 8050.6316 | 7.082817 | 6686.6982 | 1.56000 | 0.30990 | 1.600000 | 15.704552 | 0.745 | 0.492 | 29737.85 | 56.34624 |
| DZA | Algeria | 42228429 | 4114.7151 | 6.362497 | 4051.6210 | 1.83000 | 2.24000 | 1.900000 | 22.383506 | 0.759 | 0.523 | 22953.96 | 50.85326 |
| ECU | Ecuador | 17084357 | 6344.8720 | 7.157290 | 2826.0753 | 2.05000 | 1.20000 | 1.500000 | 22.383506 | 0.758 | 0.602 | 24186.41 | 42.08474 |
| EGY | Egypt | 98423595 | 2549.1323 | 5.229779 | 21946.3583 | 0.79020 | 1.39890 | 1.600000 | 30.230369 | 0.700 | 0.486 | 28086.87 | 81.81099 |
| ERI | Eritrea | 3538381 | 811.3800 | 3.950000 | 3417.0000 | 0.05570 | 0.64920 | 0.700000 | 12.562992 | 0.434 | NA | 45816.84 | NA |
| ESP | Spain | 46723749 | 30370.8923 | 19.378508 | 4676.3705 | 4.06910 | 5.53070 | 3.000000 | 18.816089 | 0.893 | 0.743 | 25224.30 | 42.45657 |
| EST | Estonia | 1320884 | 23266.3463 | 19.626357 | 2378.8761 | 3.46510 | 6.45260 | 5.000000 | 35.006568 | 0.882 | 0.747 | 34193.70 | 128.68112 |
| ETH | Ethiopia | 109224559 | 772.3135 | 3.501133 | 8321.9784 | 0.10000 | 0.84000 | 0.300000 | 10.806414 | 0.470 | 0.385 | 36767.57 | 108.23213 |
| FIN | Finland | 5518050 | 50152.3401 | 21.720788 | 1199.5169 | 3.80800 | 14.72300 | 4.400000 | 37.881170 | 0.925 | 0.814 | 29091.06 | 33.47612 |
| FJI | Fiji | 883483 | 6266.9680 | 5.449680 | 3096.5327 | 0.83740 | 2.93800 | 2.300000 | 28.251402 | 0.724 | NA | 35978.20 | NA |
| FRA | France | 66987244 | 41463.6440 | 20.034625 | 3988.0406 | 3.23490 | 9.68870 | 6.500000 | 47.511637 | 0.891 | 0.765 | 25376.30 | 34.11460 |
| FSM | Micronesia (Federated States of) | 112640 | 3568.2908 | 3.996804 | 300.0000 | 0.17700 | 3.31900 | 3.200000 | 21.507162 | 0.614 | NA | 34801.23 | NA |
| GAB | Gabon | 2119275 | 7952.5259 | 3.563907 | 2616.0560 | 0.36110 | 2.58060 | 6.300000 | 13.701733 | 0.702 | 0.453 | 36741.67 | 30.20095 |
| GBR | United Kingdom of Great Britain and Northern Ireland | 66488991 | 42943.9023 | 18.395866 | 4029.4078 | 2.80580 | 8.28780 | 2.800000 | 36.032335 | 0.920 | 0.781 | 27664.54 | 38.93694 |
| GEO | Georgia | 3731000 | 4717.1430 | 14.865491 | 2896.7173 | 5.09760 | 4.09140 | 2.600000 | 21.398766 | 0.786 | 0.614 | 38878.07 | NA |
| GHA | Ghana | 29767108 | 2202.3122 | 3.068898 | 10517.8969 | 0.18000 | 1.20000 | 0.900000 | 30.532917 | 0.596 | 0.439 | 39775.01 | 17.33792 |
| GIN | Guinea | 12414318 | 878.5996 | 2.926022 | 3256.8706 | 0.07880 | 0.38440 | 0.300000 | 12.562992 | 0.466 | 0.374 | 58109.00 | NA |
| GMB | Gambia | 2280102 | 716.1185 | 2.589981 | 4245.7841 | 0.10770 | 1.62920 | 1.100000 | 10.651398 | 0.466 | 0.397 | 39182.44 | 31.85005 |
| GNB | Guinea-Bissau | 1874309 | 777.9699 | 2.823716 | 5973.8862 | 0.20000 | 1.40000 | 1.000000 | 12.627126 | 0.461 | NA | 55151.76 | NA |
| GNQ | Equatorial Guinea | 1308974 | 10261.7600 | 2.457877 | 4588.0000 | 0.40000 | 0.50000 | 2.100000 | 9.806560 | 0.588 | NA | 37430.12 | NA |
| GRC | Greece | 10727668 | 20324.2536 | 21.655272 | 6619.4029 | 4.59200 | 3.37280 | 4.300000 | 46.550442 | 0.872 | 0.681 | 30425.98 | 57.95948 |
| GRD | Grenada | 111454 | 10640.4968 | 9.621907 | 492.0000 | 1.44710 | 3.14470 | 3.700000 | 22.383506 | 0.763 | NA | 31670.58 | NA |
| GRL | Greenland | 56025 | 48181.8700 | 9.570000 | 1250.0000 | 1.13500 | 8.59195 | 14.353400 | 37.756894 | NA | NA | 33089.18 | 63.41477 |
| GTM | Guatemala | 17247807 | 4549.0101 | 4.812073 | 5533.5684 | 0.35500 | 0.94700 | 0.600000 | 11.841197 | 0.651 | 0.460 | 28822.63 | 26.43050 |
| GUM | Guam | 165768 | 35712.5621 | 9.849911 | 1429.0000 | 1.08400 | 7.21410 | 4.300000 | 25.421083 | NA | NA | 31247.30 | 99.00314 |
| GUY | Guyana | 779004 | 4979.0022 | 6.450271 | 3654.6123 | 0.79900 | 1.33980 | 1.600000 | 22.383506 | 0.670 | 0.495 | 34812.79 | 20.54277 |
| HND | Honduras | 9587522 | 2500.1132 | 4.690618 | 1019.5430 | 0.31440 | 0.88150 | 0.700000 | 21.976087 | 0.623 | 0.490 | 24083.54 | 62.70662 |
| HRV | Croatia | 4089400 | 14909.6932 | 20.445433 | 1107.9589 | 2.99620 | 8.11340 | 5.600000 | 38.476868 | 0.837 | 0.723 | 33702.43 | 35.99865 |
| HTI | Haiti | 11123176 | 868.3420 | 4.949404 | 18010.0979 | 0.23450 | 0.68040 | 0.700000 | 12.562992 | 0.503 | 0.446 | 43644.21 | NA |
| HUN | Hungary | 9768785 | 16161.9805 | 19.157725 | 2304.7777 | 3.23120 | 6.64250 | 7.000000 | 41.601221 | 0.845 | 0.703 | 37054.77 | 50.39711 |
| IDN | Indonesia | 267663435 | 3893.5961 | 5.857166 | 6212.6763 | 0.37770 | 2.05830 | 1.200000 | 14.301779 | 0.707 | 0.535 | 28918.11 | 29.35859 |
| IMN | Man, Isle of | 84077 | 80989.1722 | 20.600000 | 2953.0000 | 1.44900 | 2.27300 | 1.584279 | 37.756894 | NA | NA | NA | NA |
| IND | India | 1352617328 | 2009.9789 | 6.179956 | 7017.7348 | 0.77760 | 2.10710 | 0.700000 | 14.862593 | 0.647 | 0.440 | 34821.51 | 28.57908 |
| IRL | Ireland | 4853506 | 78806.4320 | 13.865802 | 3419.2908 | 3.08610 | 14.29490 | 2.800000 | 23.283000 | 0.942 | 0.806 | 22827.71 | 32.83974 |
| IRN | Iran (Islamic Republic of) | 81800269 | 5627.7493 | 6.184574 | 1072.7324 | 1.14000 | 1.87000 | 1.500000 | 19.637386 | 0.797 | 0.591 | 24796.70 | 38.42209 |
| IRQ | Iraq | 38433600 | 5834.1662 | 3.323600 | 2561.6599 | 0.82170 | 1.67990 | 1.400000 | 25.303019 | 0.689 | 0.398 | 27100.99 | 63.36255 |
| ISL | Iceland | 353574 | 73191.1163 | 14.795093 | 508.3561 | 3.97010 | 15.68060 | 3.200000 | 30.775804 | 0.938 | 0.740 | 22348.55 | 32.95967 |
| ISR | Israel | 8883800 | 41715.0293 | 11.976986 | 3834.7727 | 3.21760 | 5.20440 | 3.100000 | 37.027773 | 0.906 | 0.763 | 19302.87 | 33.23459 |
| ITA | Italy | 60431283 | 34483.2040 | 22.751680 | 5121.1514 | 4.09310 | 5.86930 | 3.400000 | 41.263633 | 0.883 | 0.769 | 27217.37 | 35.57398 |
| JAM | Jamaica | 2934855 | 5354.2369 | 8.796643 | 1721.2794 | 1.31990 | 1.13970 | 1.700000 | 26.666371 | 0.726 | 0.544 | 28065.26 | 41.20042 |
| JOR | Jordan | 9956011 | 4241.7888 | 3.846490 | 4131.0094 | 2.34360 | 3.38860 | 1.400000 | 26.123615 | 0.723 | 0.562 | 19379.48 | 61.75628 |
| JPN | Japan | 126529100 | 39289.9584 | 27.576370 | 3788.8347 | 2.41180 | 11.51840 | 13.400000 | 16.441992 | 0.915 | 0.844 | 27366.88 | 17.71950 |
| KAZ | Kazakhstan | 18276499 | 9812.6011 | 7.391846 | 833.1313 | 3.25220 | 8.48900 | 6.700000 | 14.705043 | 0.817 | 0.746 | 31327.04 | 88.75176 |
| KEN | Kenya | 51393010 | 1710.5101 | 2.339187 | 15719.0314 | 0.19880 | 1.54210 | 1.400000 | 26.174526 | 0.579 | 0.518 | 36950.44 | 20.19120 |
| KGZ | Kyrgyzstan | 6315800 | 1281.3637 | 4.494728 | 3504.0806 | 1.87600 | 6.42990 | 4.500000 | 26.046652 | 0.674 | 0.580 | 26618.30 | 26.31316 |
| KHM | Cambodia | 16249798 | 1510.3249 | 4.568680 | 16916.0714 | 0.16820 | 0.95470 | 0.800000 | 14.959800 | 0.581 | 0.493 | 31981.13 | NA |
| KIR | Kiribati | 115847 | 1625.2861 | 3.952627 | 2289.2253 | 0.20280 | 4.82870 | 1.900000 | 77.360789 | 0.623 | 0.479 | 45382.34 | NA |
| KNA | Saint Kitts and Nevis | 52441 | 19275.4185 | 9.030000 | 757.0000 | 2.52300 | 3.97790 | 2.300000 | 24.305378 | 0.777 | NA | NA | NA |
| KOR | Republic of Korea | 51635256 | 31362.7515 | 14.418556 | 7690.2882 | 2.36610 | 6.97350 | 11.500000 | 25.421083 | 0.906 | 0.845 | 22538.36 | 53.42279 |
| KWT | Kuwait | 4137309 | 33994.4066 | 2.550472 | 6493.4568 | 2.57890 | 6.96940 | 2.000000 | 51.999664 | 0.808 | 0.576 | 16869.47 | 33.37819 |
| LAO | Lao People’s Democratic Republic | 7061507 | 2542.4865 | 4.075150 | 2420.6386 | 0.49970 | 0.97560 | 1.500000 | 22.383506 | 0.604 | 0.452 | 36844.85 | 61.69925 |
| LBN | Lebanon | 6848925 | 8269.7877 | 7.002368 | 31341.1887 | 2.27100 | 2.64480 | 2.900000 | 25.805421 | 0.730 | 0.538 | 21119.88 | 33.31959 |
| LBR | Liberia | 4818977 | 677.3222 | 3.253432 | 3223.5510 | 0.03730 | 0.10070 | 0.800000 | 16.484887 | 0.465 | 0.319 | 45781.67 | 59.09309 |
| LBY | Libya | 6678567 | 7241.7045 | 4.392040 | 1553.0000 | 2.15810 | 6.74160 | 3.700000 | 22.383506 | 0.708 | NA | 27628.01 | 116.01993 |
| LCA | Saint Lucia | 181889 | 10566.0498 | 9.806530 | 1815.9384 | 0.10530 | 1.58730 | 1.300000 | 19.373038 | 0.745 | NA | 28439.89 | NA |
| LIE | Liechtenstein | 37910 | 165028.2500 | 17.910000 | 755.0000 | 3.46510 | 8.59195 | 4.700000 | 37.756894 | 0.917 | NA | NA | NA |
| LKA | Sri Lanka | 21670000 | 4102.4814 | 10.473220 | 7090.8370 | 0.95800 | 2.11550 | 3.600000 | 16.238911 | 0.780 | 0.584 | 23936.38 | 29.09383 |
| LSO | Lesotho | 2108132 | 1299.1531 | 4.901087 | 1855.4433 | 0.06760 | 0.65080 | 1.300000 | 35.237078 | 0.518 | 0.371 | 67300.93 | NA |
| LTU | Lithuania | 2789533 | 19153.4090 | 19.705033 | 1154.8017 | 4.33700 | 7.91550 | 7.300000 | 31.108741 | 0.869 | 0.712 | 41149.50 | NA |
| LUX | Luxembourg | 607728 | 116639.8880 | 14.183154 | 2294.6793 | 3.02660 | 12.34960 | 4.800000 | 38.742076 | 0.909 | 0.692 | 25319.30 | 68.03818 |
| LVA | Latvia | 1926542 | 17860.6172 | 20.043620 | 2657.0803 | 3.19460 | 4.81550 | 5.800000 | 43.015702 | 0.854 | 0.724 | 40947.22 | NA |
| MAC | Macau | 631636 | 87208.5359 | 10.484203 | 72754.2891 | 1.56200 | 7.21410 | 5.295300 | 15.075829 | NA | 0.759 | NA | NA |
| MAR | Morocco | 36029138 | 3237.8834 | 7.012905 | 8277.7186 | 0.72730 | 1.09790 | 1.100000 | 24.683407 | 0.676 | 0.500 | 29151.04 | 52.62724 |
| MCO | Monaco | 38682 | 185741.2800 | 33.150000 | 20629.6758 | 6.56170 | 20.26250 | 13.800000 | 37.756894 | NA | NA | NA | NA |
| MDA | Republic of Moldova | 3545883 | 3227.3122 | 11.469556 | 1193.6244 | 3.20020 | 4.51130 | 5.800000 | 27.319027 | 0.711 | 0.580 | 38891.64 | 46.71659 |
| MDG | Madagascar | 26262368 | 527.5013 | 2.986717 | 6923.3060 | 0.18120 | 0.10590 | 0.200000 | 9.798578 | 0.521 | 0.374 | 48534.17 | 73.77367 |
| MDV | Maldives | 515696 | 10330.6156 | 3.703345 | 1000.0000 | 1.03790 | 3.95040 | 4.300000 | 25.062388 | 0.719 | NA | 15334.39 | NA |
| MEX | Mexico | 126190788 | 9673.4437 | 7.223685 | 8150.6268 | 2.24780 | 2.90000 | 1.500000 | 20.449238 | 0.767 | 0.607 | 25259.42 | 26.79982 |
| MHL | Marshall Islands | 58413 | 3788.1636 | 4.240000 | 1398.1362 | 0.45540 | 3.54840 | 2.700000 | 57.865036 | 0.698 | NA | 37806.23 | NA |
| MKD | Republic of North Macedonia | 2082958 | 6083.7189 | 13.670181 | 1100.0000 | 2.87360 | 3.79170 | 4.400000 | 27.869086 | 0.759 | 0.534 | 30829.17 | NA |
| MLI | Mali | 19077690 | 899.6599 | 2.507230 | 15180.5143 | 0.13930 | 0.38200 | 0.100000 | 12.498858 | 0.427 | 0.317 | 69095.54 | 103.69007 |
| MLT | Malta | 483530 | 30098.2833 | 20.349324 | 3134.4172 | 3.82600 | 8.95000 | 4.700000 | 34.915014 | 0.885 | 0.701 | 28125.93 | NA |
| MMR | Myanmar | 53708395 | 1325.9529 | 5.784642 | 7505.3126 | 0.86400 | 0.97940 | 0.900000 | 16.750981 | 0.584 | 0.473 | 35568.82 | 28.78576 |
| MNE | Montenegro | 622345 | 8844.2370 | 14.974937 | 2164.0000 | 2.33370 | 5.71790 | 4.000000 | 28.940219 | 0.816 | 0.615 | 32166.37 | NA |
| MNG | Mongolia | 3170208 | 4121.7324 | 4.083539 | 2406.5120 | 2.88660 | 3.98460 | 7.000000 | 23.815795 | 0.735 | 0.626 | 33454.82 | 46.27480 |
| MNP | Northern Mariana Islands | 56882 | 23258.6759 | 5.980000 | 1607.3727 | 0.44500 | 7.21410 | 4.300000 | 25.421083 | NA | NA | 27138.94 | NA |
| MOZ | Mozambique | 29495962 | 498.9572 | 2.890764 | 4253.0618 | 0.07350 | 0.44360 | 0.700000 | 0.020051 | 0.446 | 0.361 | 56426.69 | 32.65729 |
| MRT | Mauritania | 4403319 | 1188.8346 | 3.141112 | 6643.5977 | 0.17900 | 1.03410 | 0.400000 | 22.383506 | 0.527 | 0.353 | 31474.58 | 26.90134 |
| MUS | Mauritius | 1265303 | 11238.6904 | 11.474173 | 3510.2040 | 2.02460 | 3.38310 | 3.400000 | 23.267832 | 0.796 | 0.626 | 31645.39 | 94.47944 |
| MWI | Malawi | 18143315 | 389.3980 | 2.645435 | 5751.7148 | 0.01570 | 0.25280 | 1.300000 | 20.865540 | 0.485 | 0.407 | 48137.77 | 26.87233 |
| MYS | Malaysia | 31528585 | 11373.2330 | 6.671755 | 2917.8093 | 1.51320 | 4.07180 | 1.900000 | 15.931363 | 0.804 | 0.622 | 23253.37 | 39.81256 |
| NAM | Namibia | 2448255 | 5931.4539 | 3.636032 | 4045.0419 | 0.37400 | 2.77500 | 2.700000 | 31.122577 | 0.645 | 0.435 | 39432.37 | 29.12585 |
| NCL | New Caledonia | 284060 | 12579.6000 | 9.174158 | 2203.3447 | 1.98200 | 7.21410 | 4.300000 | 25.421083 | NA | NA | NA | NA |
| NER | Niger | 22442948 | 413.9803 | 2.595008 | 3646.1203 | 0.05000 | 0.31090 | 0.300000 | 10.164529 | 0.377 | 0.316 | 61357.01 | 67.67389 |
| NGA | Nigeria | 195874740 | 2028.1820 | 2.747377 | 6064.4800 | 0.38270 | 1.45240 | 0.500000 | 18.478845 | 0.534 | 0.342 | 57098.05 | 37.91898 |
| NIC | Nicaragua | 6465513 | 2028.8948 | 5.247497 | 3372.0485 | 1.00560 | 1.57750 | 0.900000 | 16.728304 | 0.651 | 0.531 | 20145.23 | 14.31593 |
| NLD | Netherlands | 17231017 | 53024.0592 | 19.196193 | 2473.9182 | 3.50670 | 11.10430 | 4.700000 | 37.756894 | 0.934 | 0.800 | 26962.03 | 58.29695 |
| NOR | Norway | 5314336 | 81697.2460 | 17.049222 | 967.4282 | 4.63360 | 18.12470 | 3.900000 | 37.703024 | 0.954 | 0.771 | 25119.19 | 40.47480 |
| NPL | Nepal | 28087871 | 1033.9124 | 5.727671 | 30395.6367 | 0.65070 | 2.68540 | 0.300000 | 19.377208 | 0.579 | 0.490 | 30160.12 | NA |
| NRU | Nauru | 12704 | 9888.8936 | 2.860000 | 1260.0620 | 1.23890 | 6.19470 | 5.000000 | 84.972358 | NA | NA | NA | NA |
| NZL | New Zealand | 4885500 | 41945.3317 | 15.652425 | 2419.3986 | 3.02520 | 10.95500 | 2.800000 | 30.686560 | 0.921 | 0.767 | 26137.73 | 52.18328 |
| OMN | Oman | 4829483 | 16415.1573 | 2.392787 | 921.0000 | 1.96970 | 4.30040 | 1.600000 | 27.968638 | 0.834 | 0.622 | 19631.57 | 34.86040 |
| PAK | Pakistan | 212215030 | 1482.4031 | 4.312774 | 9458.6742 | 0.97530 | 0.50040 | 0.600000 | 23.631328 | 0.560 | 0.389 | 39350.07 | 43.47958 |
| PAN | Panama | 4176873 | 15575.0726 | 8.104731 | 6338.6782 | 1.56990 | 1.41000 | 2.300000 | 29.647642 | 0.795 | 0.532 | 22459.42 | 30.68192 |
| PER | Peru | 31989256 | 6941.2358 | 8.088393 | 6973.2788 | 1.27000 | 1.35000 | 1.600000 | 20.319493 | 0.759 | 0.586 | 21116.53 | 75.95754 |
| PHL | Philippines | 106651922 | 3102.7134 | 5.122569 | 11239.5361 | 1.27500 | 0.24000 | 1.000000 | 14.896826 | 0.712 | 0.548 | 31452.21 | 48.42471 |
| PLW | Palau | 17907 | 15859.4349 | 8.500000 | 143.6626 | 1.18480 | 5.26070 | 4.800000 | 41.358824 | 0.814 | NA | NA | NA |
| PNG | Papua New Guinea | 8606316 | 2730.2748 | 3.445269 | 1453.8555 | 0.05290 | 0.51250 | 4.024200 | 19.059287 | 0.543 | 0.384 | 52603.89 | 36.81704 |
| POL | Poland | 37978548 | 15420.9112 | 17.517817 | 2254.9327 | 2.39980 | 5.72210 | 6.500000 | 34.217365 | 0.872 | 0.747 | 32741.56 | 30.03679 |
| PRI | Puerto Rico | 3195153 | 31651.3482 | 18.674850 | 5035.0459 | 1.75000 | 3.12810 | 3.320000 | 29.647642 | NA | NA | 30737.06 | 64.74040 |
| PRK | North Korea | 25549819 | 1700.0000 | 9.334520 | 1453.8365 | 3.67450 | 4.43820 | 13.200000 | 12.562992 | NA | NA | 33645.72 | NA |
| PRT | Portugal | 10281762 | 23407.9066 | 21.953858 | 6068.3332 | 3.33560 | 6.37250 | 3.400000 | 41.448742 | 0.850 | 0.776 | 29488.43 | 40.24497 |
| PRY | Paraguay | 6956071 | 5821.8143 | 6.430215 | 4219.4013 | 1.36600 | 1.16690 | 1.300000 | 15.622963 | 0.724 | 0.533 | 23315.58 | 41.89047 |
| PSE | West Bank | 4569087 | 3198.8666 | 3.133306 | 6092.4053 | 0.83700 | 2.63510 | 1.200000 | 8.305154 | 0.690 | 0.554 | 20265.41 | 55.71990 |
| PYF | French Polynesia | 277679 | 14323.8200 | 8.290508 | 1425.7722 | 1.69700 | 7.21410 | 4.300000 | 25.421083 | NA | NA | NA | NA |
| QAT | Qatar | 2781677 | 68793.7844 | 1.370070 | 14026.9244 | 0.00080 | 6.60280 | 1.200000 | 27.968638 | 0.848 | 0.615 | 16311.49 | 27.25396 |
| RKS | Kosovo | 1845300 | 4302.2765 | 7.430000 | 2610.3152 | 2.89900 | 5.71790 | 4.700000 | 28.940219 | NA | NA | NA | NA |
| ROU | Romania | 19473936 | 12301.1864 | 18.338701 | 3235.8430 | 2.25860 | 6.09790 | 6.300000 | 31.591276 | 0.816 | 0.601 | 38896.25 | 25.15149 |
| RUS | Russian Federation | 144478050 | 11288.8724 | 14.674708 | 1445.6886 | 4.01390 | 8.62120 | 8.200000 | 28.940219 | 0.824 | 0.729 | 41883.81 | 34.10561 |
| RWA | Rwanda | 12301939 | 772.9445 | 2.938196 | 7578.3369 | 0.13460 | 0.83070 | 1.600000 | 18.903174 | 0.536 | 0.374 | 35325.34 | 42.99572 |
| SAU | Saudi Arabia | 33699947 | 23338.9635 | 3.314088 | 1389.0000 | 2.39000 | 5.70000 | 2.700000 | 27.968638 | 0.857 | 0.585 | 20148.10 | 42.39795 |
| SDN | Sudan | 41801533 | 977.2736 | 3.581161 | 1407.7720 | 0.41000 | 0.83000 | 0.800000 | 10.477965 | 0.508 | 0.379 | 35526.64 | 30.31861 |
| SEN | Senegal | 15854360 | 1521.9536 | 3.086824 | 9336.1025 | 0.06920 | 0.31290 | 0.300000 | 18.298812 | 0.514 | 0.418 | 36347.16 | 44.42331 |
| SGP | Singapore | 5638676 | 64581.9440 | 11.463380 | 7815.7468 | 2.30630 | 7.21410 | 2.400000 | 15.205542 | 0.935 | 0.884 | 17207.64 | 44.55065 |
| SLB | Solomon Islands | 652858 | 2137.6907 | 3.598950 | 4251.8244 | 0.19990 | 2.13070 | 1.400000 | 31.921564 | 0.557 | 0.443 | 35356.97 | NA |
| SLE | Sierra Leone | 7650154 | 533.9912 | 2.966556 | 11207.5462 | 0.02500 | 0.99620 | 0.400000 | 12.562992 | 0.438 | 0.351 | 61567.32 | 53.95067 |
| SLV | El Salvador | 6420744 | 4058.2524 | 8.287090 | 3906.2752 | 1.56900 | 2.27800 | 1.300000 | 24.895007 | 0.667 | 0.501 | 28276.20 | 45.12273 |
| SMR | San Marino | 33785 | 48494.5514 | 19.800000 | 1195.0000 | 6.14680 | 8.77680 | 3.800000 | 42.198874 | NA | NA | NA | 59.16167 |
| SOM | Somalia | 15008154 | 314.5442 | 2.873311 | 2863.8412 | 0.02290 | 0.06110 | 0.900000 | 0.000237 | NA | NA | 58321.04 | 52.37422 |
| SRB | Serbia | 6982084 | 7246.7317 | 18.345793 | 3148.0612 | 3.12500 | 6.12030 | 5.700000 | 36.893403 | 0.799 | 0.755 | 36971.30 | 22.44401 |
| SSD | South Sudan | 10975920 | 1119.6500 | 3.398483 | 5000.0000 | 0.06290 | 0.44360 | 0.700000 | 12.562992 | 0.413 | 0.302 | 71068.46 | 34.94162 |
| STP | Sao Tome and Principe | 211028 | 2001.1409 | 2.925678 | 8850.2393 | 0.32000 | 2.26000 | 2.900000 | 12.562992 | 0.609 | NA | 29094.90 | NA |
| SUR | Suriname | 575991 | 6234.0449 | 6.906370 | 1465.1056 | 1.22650 | 4.09830 | 3.100000 | 12.562992 | 0.724 | NA | 32526.55 | 85.84998 |
| SVK | Slovakia | 5447011 | 19442.7058 | 15.629247 | 2263.7493 | 2.46430 | 9.17270 | 5.800000 | 12.562992 | 0.857 | 0.694 | 32009.70 | 24.65666 |
| SVN | Slovenia | 2067372 | 26123.9739 | 19.606880 | 1989.1859 | 2.99530 | 9.68000 | 4.600000 | 12.562992 | 0.902 | 0.788 | 30107.05 | 64.55297 |
| SWE | Sweden | 10183175 | 54608.3602 | 20.095525 | 3531.1422 | 5.39960 | 11.54340 | 2.600000 | 12.562992 | 0.937 | 0.800 | 26039.04 | 44.21572 |
| SWZ | Eswatini | 1136191 | 4145.9741 | 4.014588 | 1053.5995 | 0.07960 | 2.00000 | 2.100000 | 12.562992 | 0.608 | 0.409 | 51280.62 | 42.09531 |
| SYC | Seychelles | 96762 | 16433.9353 | 7.594546 | 1074.7065 | 0.94580 | 3.26130 | 3.600000 | 12.562992 | 0.801 | 0.678 | 29181.51 | 18.93996 |
| SYR | Syrian Arab Republic | 16906283 | 2032.6200 | 4.503124 | 5413.9058 | 1.22000 | 1.46000 | 1.500000 | 12.562992 | 0.549 | NA | 35593.37 | 21.00999 |
| TCA | Turks and Caicos Islands | 37665 | 27142.2278 | 4.760000 | 1022.4028 | 1.93815 | 3.12810 | 2.900000 | 12.562992 | NA | NA | NA | NA |
| TCD | Chad | 15477751 | 728.3432 | 2.480519 | 2021.5796 | 0.04750 | 0.36370 | 0.400000 | 12.562992 | 0.401 | 0.293 | 71486.42 | 74.98054 |
| TGO | Togo | 7889094 | 679.2571 | 2.869468 | 5392.5977 | 0.04870 | 0.29800 | 0.700000 | 17.886013 | 0.513 | 0.413 | 43432.93 | NA |
| THA | Thailand | 69428524 | 7273.5632 | 11.900893 | 8827.4164 | 0.80960 | 2.96470 | 2.100000 | 18.478845 | 0.765 | 0.604 | 27655.14 | 69.79101 |
| TJK | Tajikistan | 9100837 | 826.6215 | 3.021888 | 6491.1939 | 1.70030 | 5.23300 | 4.800000 | 13.747442 | 0.656 | 0.533 | 31468.63 | NA |
| TKM | Turkmenistan | 5850908 | 6966.6354 | 4.426634 | 3128.8886 | 2.22480 | 4.63060 | 7.400000 | 28.940219 | 0.710 | NA | 31282.38 | NA |
| TLS | Timor-Leste | 1267972 | 2035.5339 | 4.316492 | 8946.4346 | 0.71970 | 1.67010 | 5.900000 | 36.356229 | 0.626 | 0.434 | 30155.03 | NA |
| TON | Tonga | 103197 | 4364.0156 | 5.953661 | 288.8070 | 0.52230 | 3.93200 | 2.600000 | 31.401971 | 0.717 | 0.512 | 28461.62 | NA |
| TTO | Trinidad and Tobago | 1389858 | 17129.9131 | 10.734954 | 1675.0000 | 2.66970 | 3.50930 | 3.000000 | 35.928554 | 0.799 | 0.613 | 32094.99 | 32.69817 |
| TUN | Tunisia | 11565204 | 3447.5079 | 8.315679 | 4330.2120 | 1.27220 | 2.63510 | 2.300000 | 35.463454 | 0.739 | 0.508 | 23924.84 | 76.62440 |
| TUR | Turkey | 82319724 | 9370.1763 | 8.483213 | 4156.1038 | 1.76050 | 2.63040 | 2.700000 | 33.872430 | 0.807 | 0.626 | 23498.33 | 24.26670 |
| TUV | Tuvalu | 11508 | 3700.7107 | 6.280000 | 1316.8639 | 0.91740 | 3.76150 | 5.600000 | 28.251402 | NA | 0.554 | NA | NA |
| TZA | United Republic of Tanzania | 56318348 | 1050.6753 | 2.601299 | 10824.9011 | 0.03990 | 0.41260 | 0.700000 | 14.324209 | 0.528 | 0.400 | 42037.92 | 43.04168 |
| UGA | Uganda | 42723139 | 642.7767 | 1.940987 | 7829.3496 | 0.09080 | 0.63030 | 0.500000 | 14.193050 | 0.528 | 0.382 | 42552.68 | 24.17427 |
| UKR | Ukraine | 44622516 | 3095.1736 | 16.434686 | 1976.0174 | 3.00750 | 7.05760 | 8.800000 | 33.423702 | 0.750 | 0.647 | 47426.96 | 65.52201 |
| URY | Uruguay | 3449299 | 17277.9701 | 14.814520 | 5556.2719 | 5.04990 | 1.92990 | 2.800000 | 34.989907 | 0.808 | 0.600 | 29758.62 | 118.12991 |
| USA | United States of America | 327167434 | 62794.5857 | 15.807654 | 1719.9951 | 2.59480 | 8.55000 | 2.900000 | 22.367648 | 0.920 | 0.762 | 30958.02 | 26.82018 |
| UZB | Uzbekistan | 32955400 | 1532.3717 | 4.419138 | 14209.0000 | 2.36850 | 12.07390 | 4.000000 | 17.089524 | 0.710 | NA | 29255.64 | 39.64021 |
| VCT | Saint Vincent and the Grenadines | 110210 | 7361.4010 | 9.589787 | 1998.8293 | 0.65870 | 2.58010 | 2.600000 | 26.350707 | 0.728 | NA | 33096.44 | NA |
| VEN | Venezuela (Bolivarian Republic of) | 28870195 | 16054.4900 | 7.266987 | 1619.5830 | 1.92400 | 1.12230 | 0.800000 | 22.383506 | 0.726 | NA | 26598.25 | 56.34925 |
| VIR | Virgin Islands | 106977 | 35938.0244 | 19.288307 | 1221.4246 | 1.64500 | 3.12810 | 18.680000 | 29.647642 | NA | NA | 37279.79 | NA |
| VNM | Viet Nam | 95540395 | 2566.5970 | 7.274978 | 14689.0954 | 0.81990 | 1.43210 | 2.600000 | 21.945334 | 0.693 | 0.666 | 25809.19 | 38.91008 |
| VUT | Vanuatu | 292680 | 3123.8934 | 3.640153 | 2610.3152 | 0.17060 | 1.38530 | 1.700000 | 25.632959 | 0.597 | 0.471 | 38792.10 | NA |
| WSM | Western Samoa | 196130 | 4183.4079 | 4.800412 | 676.0000 | 0.34090 | 1.85650 | 1.000000 | 27.786397 | 0.707 | NA | 25162.69 | NA |
| YEM | Yemen | 28498687 | 944.4085 | 2.876270 | 10631.8888 | 0.31040 | 0.73000 | 0.700000 | 12.562992 | 0.463 | 0.369 | 38986.96 | 57.56986 |
| ZAF | South Africa | 57779622 | 6374.0282 | 5.318005 | 4269.1238 | 0.91010 | 3.51710 | 2.800000 | 34.416834 | 0.705 | 0.406 | 43715.12 | 19.61127 |
| ZMB | Zambia | 17351822 | 1539.9002 | 2.099678 | 2712.4236 | 0.09130 | 0.89250 | 2.000000 | 20.808263 | 0.591 | 0.396 | 45609.19 | 49.65782 |
| ZWE | Zimbabwe | 14439018 | 2146.9964 | 2.939524 | 2406.2843 | 0.07630 | 1.15460 | 1.700000 | 26.596372 | 0.563 | 0.441 | 47439.85 | 32.72253 |

# 3 Covid -19 Regression Model Function

```
setwd("D:/VS/COVIDModel")
covid19_RegModel <- function(SI.date, df_c, df_d, df_r, BaseData_ss, si_source, CountryCodeList, iv_norm, iv_rev, dv, normalization) {
    tryCatch({

        # Stringency
        si <- si_source[c("country_code", SI.date)]
        colnames(si) <- c("country_code", "stringency")

        #-----CovidCases

        data_Cov_ss <- covid_data_day(SI.date, df_c, df_d, df_r)
        data_Cov_ss_g <- covid_data_day_growth(SI.date, df_c, df_d, df_r)

        colnames(CountryCodeList) <- c("country", "country_code")
        data_Cov_ss1 <- merge(data_Cov_ss, CountryCodeList, by = 'country', all.x = T)

        data_Cov_ss2 <- data.frame(country_code = data_Cov_ss1$country_code, deaths = data_Cov_ss1$deaths,
                           confirmed = data_Cov_ss1$confirmed, active = data_Cov_ss1$active,
                           deathsGrowth = data_Cov_ss_g$deathsGrowth,
                           confirmedGrowth = data_Cov_ss_g$confirmedGrowth,
                           recoveredGrowth = data_Cov_ss_g$recoveredGrowth)
        #----- Data Integration
        data_m_si <- merge(si, BaseData_ss, by = "country_code", all.x = T)
        data_m <- merge(data_m_si, data_Cov_ss2, by = "country_code", all.x = T)
        maximumPercent <- data_m$confirmed / data_m$Pop_2018
        
        #data_m$susceptibles = (data_m$Pop_2018 - data_m$confirmed) * 100 / data_m$Pop_2018
        data_m$susceptibles = ((data_m$Pop_2018 * max(na.omit(maximumPercent)))- data_m$confirmed) / (data_m$Pop_2018 * max(na.omit(maximumPercent)))
        data_m$deaths = (data_m$deaths) / data_m$Pop_2018 * 1000000
        data_m$confirmed = (data_m$confirmed) / data_m$Pop_2018 * 1000000
        data_m$active = (data_m$active) / data_m$Pop_2018 * 1000000
        

        #################
        if (normalization == "BoxCox") {
            #---------- Weight Model Preparation 
            data_num <- sapply(data_m[, 2:ncol(data_m)], as.numeric)
            data_num <- cbind(data_m[, 1:2], data_num)
            data_a <- data_num[apply(data_num[, 2:ncol(data_num)] > 0, 1, all),]
            data_a <- na.omit(data_a)
            data_a <- data_a[, -3]

            #--Split Tab for normalization

            data_sp <- data_a[, 2:ncol(data_a)]
            data_sp <- sapply(data_sp, as.numeric)

            #-- Normalize Data

            data_normal <- boxcox(data_sp)
            data_normal <- as.data.frame(data_normal$x.t)
            data_normal <- cbind(data_a["country_code"], data_normal)

        }
        ############################

        ##########################
        if (normalization == "Scaling") {
            data_num <- sapply(data_m[, 2:ncol(data_m)], as.numeric)
            

            #Normalize Data
            data_normal <- scale(data_num)
            data_normal <- cbind(data_m["country_code"], data_normal)
        }
        ##########################

        #iv = append(iv_norm, iv_rev)
        iv = iv_norm
        #str(data_normal)
        df_dv = data.frame(y = data_normal[, dv])
        df_iv = data.frame(data_normal[, iv])
        df_model = cbind(df_dv, df_iv)

        model <- lm(y ~ ., data = df_model)
        model_sum <- summary(model)

        #Model VIF
        model_vif <- car::vif(model)
        
        model_weights <- calc.relimp(model, type = c("lmg"), rela = TRUE)
        #model_weights@lmg

        

        model_results <- list(model_sum, model_weights, model_vif)
        #------- Binning with Weights

        return(model_results)
    },
    error = function(err) {
        return(list(syn = word))
    })
}
```

# 4 Pre-COVID19 - Pairwise Risk Assessment

## 4.1 Pairwise Risk Evaluation Function

```
setwd("D:/VS/COVIDModel")
covid19RiskEvaluation_pairwise <- function(SI.date, BaseData_ss, CountryCodeList, iv_norm, iv_rev, imp) {
    tryCatch({

        #Normalize Data
        data_m <- BaseData_ss
        data_num <- sapply(data_m[, 2:ncol(data_m)], as.numeric)
        data_normal <- scale(data_num)
        #data_normal <- as.data.frame(data_normal$x.t)
        data_normal <- cbind(data_m["country_code"], data_normal)

        #------- Binning with Weights

        for (i in iv_norm) {
            #if (colnames(data_normal[i]) == "confirmed") {
            data_normal[i] <- cut(as.numeric(unlist(data_normal[i])), 10, labels = c(1, 2, 3, 4, 5, 6, 7, 8, 9, 10))
            data_normal[i] <- as.numeric(unlist(data_normal[i])) * as.numeric(imp[i])
        }

        for (i in iv_rev) {
            #if (colnames(data_normal[i]) == "confirmed") {
            data_normal[i] <- cut(as.numeric(unlist(data_normal[i])), 10, labels = c(10, 9, 8, 7, 6, 5, 4, 3, 2, 1))
            data_normal[i] <- as.numeric(unlist(data_normal[i])) * as.numeric(imp[i])
        }

        # --Risk Aggregation

        RiskVariables <- append(c("country_code"), iv_norm)
        RiskVariables <- append(RiskVariables, iv_rev)

        data_Risk <- data_normal[RiskVariables]
        data_Risk <- mutate(data_Risk, RiskValue = rowSums(data_Risk[, -1], na.rm = TRUE))

        data_Risk <- transform(data_Risk, RiskValue = as.numeric(unlist(RiskValue)))
        #data_Risk_ss <- data_Risk[, c(1, 2, 3, 4, 5, 6, 7, 8, 9, 10, 16, 17)]

        data_Risk$RiskRank <- cut(data_Risk[, "RiskValue"], 10, labels = c(1, 2, 3, 4, 5, 6, 7, 8, 9, 10))

        Risk <- data_Risk[c("country_code", "RiskValue", "RiskRank")]
        colnames(Risk) <- c("country_code", str_c("RiskValue_", SI.date), str_c("RiskRank_", SI.date))
        return(Risk)
    },
    error = function(err) {
        return(list(syn = word))
    })
}
```

## 4.2 Pre-COVID19 Weights Extraction

```
setwd("D:/VS/COVIDModel")

A = t(matrix(c(1, 1/2, 1/3, 1/4, 1/5, 1/5, 1/5, 1/5, 1/5, 2, 1, 1/2, 1/3, 1/4, 1/4, 1/4, 1/4, 1/4, 3,
    2, 1, 1/2, 1/3, 1/3, 1/3, 1/3, 1/3, 4, 3, 2, 1, 1/2, 1/2, 1/2, 1/2, 1/2, 5, 4, 3, 2, 1,
    1, 1, 1, 1, 5, 4, 3, 2, 1, 1, 1, 1, 1, 5, 4, 3, 2, 1, 1, 1, 1, 1, 5, 4, 3, 2, 1, 1, 1, 1, 1,
    5, 4, 3, 2, 1, 1, 1, 1, 1), nrow = 9, ncol = 9))

colnames(A) = c("PopDen_Avg", "Pop_2018", "Expenditure", "GDP_PC", "DALYs", "Nurses", "Physician", "HospitalBeds", "A65abp_2018")
rownames(A) = c("PopDen_Avg", "Pop_2018", "Expenditure", "GDP_PC", "DALYs", "Nurses", "Physician", "HospitalBeds", "A65abp_2018"); 


B = t(matrix(c(1, 1 / 2, 1 / 3, 1 / 4, 1 / 5, 1 / 5, 1 / 5, 1 / 5, 1 / 5, 2, 1, 1 / 2, 1 / 3, 1 / 4, 1 / 4, 1 / 4, 1 / 4, 1 / 4, 3,
    2, 1, 1 / 2, 1 / 3, 1 / 3, 1 / 3, 1 / 3, 1 / 3, 4, 3, 2, 1, 1 / 2, 1 / 2, 1 / 2, 1 / 2, 1 / 2, 5, 4, 3, 2, 1,
    1, 1, 1, 1, 5, 4, 3, 2, 1, 1, 1, 1, 1, 5, 4, 3, 2, 1, 1, 1, 1, 1, 5, 4, 3, 2, 1, 1, 1, 1, 1,
    5, 4, 3, 2, 1, 1, 1, 1, 1), nrow = 9, ncol = 9))

colnames(B) = c("PopDen_Avg", "Pop_2018", "Expenditure", "GDP_PC", "DALYs", "Nurses", "Physician", "HospitalBeds", "A65abp_2018")
rownames(B) = c("PopDen_Avg", "Pop_2018", "Expenditure", "GDP_PC", "DALYs", "Nurses", "Physician", "HospitalBeds", "A65abp_2018")


criteria = matrix(c(1, 1,1,1), nrow = 2, ncol = 2)

colnames(criteria) = c("A", "B")
rownames(criteria) = c("A", "B")


alternatives = list(A, B)
weight = AHP(criteria, alternatives)
impPre <- weight

kable(A) %>%
     kable_styling("striped", full_width = F, fixed_thead = T) %>%
     row_spec(0) %>%
     scroll_box(width = "910px", height = "440px")
```

|  | PopDen\_Avg | Pop\_2018 | Expenditure | GDP\_PC | DALYs | Nurses | Physician | HospitalBeds | A65abp\_2018 |
| --- | --- | --- | --- | --- | --- | --- | --- | --- | --- |
| PopDen\_Avg | 1 | 0.5 | 0.3333333 | 0.2500000 | 0.2000000 | 0.2000000 | 0.2000000 | 0.2000000 | 0.2000000 |
| Pop\_2018 | 2 | 1.0 | 0.5000000 | 0.3333333 | 0.2500000 | 0.2500000 | 0.2500000 | 0.2500000 | 0.2500000 |
| Expenditure | 3 | 2.0 | 1.0000000 | 0.5000000 | 0.3333333 | 0.3333333 | 0.3333333 | 0.3333333 | 0.3333333 |
| GDP\_PC | 4 | 3.0 | 2.0000000 | 1.0000000 | 0.5000000 | 0.5000000 | 0.5000000 | 0.5000000 | 0.5000000 |
| DALYs | 5 | 4.0 | 3.0000000 | 2.0000000 | 1.0000000 | 1.0000000 | 1.0000000 | 1.0000000 | 1.0000000 |
| Nurses | 5 | 4.0 | 3.0000000 | 2.0000000 | 1.0000000 | 1.0000000 | 1.0000000 | 1.0000000 | 1.0000000 |
| Physician | 5 | 4.0 | 3.0000000 | 2.0000000 | 1.0000000 | 1.0000000 | 1.0000000 | 1.0000000 | 1.0000000 |
| HospitalBeds | 5 | 4.0 | 3.0000000 | 2.0000000 | 1.0000000 | 1.0000000 | 1.0000000 | 1.0000000 | 1.0000000 |
| A65abp\_2018 | 5 | 4.0 | 3.0000000 | 2.0000000 | 1.0000000 | 1.0000000 | 1.0000000 | 1.0000000 | 1.0000000 |

```
weight <- data.frame(weight)

kable(weight) %>%
     kable_styling("striped", full_width = F, fixed_thead = T) %>%
     row_spec(0) %>%
     scroll_box(width = "910px", height = "440px")
```

|  | weight |
| --- | --- |
| PopDen\_Avg | 0.0270130 |
| Pop\_2018 | 0.0383154 |
| Expenditure | 0.0575581 |
| GDP\_PC | 0.0905107 |
| DALYs | 0.1573205 |
| Nurses | 0.1573205 |
| Physician | 0.1573205 |
| HospitalBeds | 0.1573205 |
| A65abp\_2018 | 0.1573205 |

```
print(str_c("Consistancy Ratio:  ", round(pairwiseConsistencyMeasures(A)$CR, 3)))
```

```
## [1] "Consistancy Ratio:  0.007"
```

## 4.3 Pre-COVID-19 Risk Evaluation

```
    setwd("D:/VS/COVIDModel")
    PreCOVID19_Risk <- covid19RiskEvaluation_pairwise(SI.date = "2020-01-01",
    BaseData_ss = BaseData[, c(1, 3:13)],
    CountryCodeList <- read.csv("./data/country_JHU.csv", stringsAsFactors = FALSE, check.names = FALSE, fileEncoding = "UTF-8-BOM"),
    iv_norm = c("DALYs", "Pop_2018", "A65abp_2018", "PopDen_Avg"),
    iv_rev = c("GDP_PC", "Physician", "Nurses", "HospitalBeds", "Expenditure"),
    imp = impPre)
```

## 4.4 Pre-COVID-19 Risk Results

```
kable(PreCOVID19_Risk) %>%
     kable_styling("striped", full_width = F, fixed_thead = T) %>%
     row_spec(0) %>%
     scroll_box(width = "910px", height = "440px")
```

| country\_code | RiskValue\_2020-01-01 | RiskRank\_2020-01-01 |
| --- | --- | --- |
| ABW | 2.049788 | 2 |
| AFG | 1.859515 | 1 |
| AGO | 1.740866 | 1 |
| ALB | 2.216360 | 2 |
| AND | 3.141780 | 4 |
| ARE | 1.967624 | 2 |
| ARG | 2.845642 | 3 |
| ARM | 2.845642 | 3 |
| ASM | 1.801956 | 1 |
| ATG | 2.431239 | 2 |
| AUS | 3.961335 | 5 |
| AUT | 4.615223 | 6 |
| AZE | 3.060521 | 4 |
| BDI | 1.771411 | 1 |
| BEL | 4.243024 | 6 |
| BEN | 1.686840 | 1 |
| BFA | 2.059039 | 2 |
| BGD | 1.464541 | 1 |
| BGR | 4.004444 | 5 |
| BHR | 1.731615 | 1 |
| BHS | 2.149550 | 2 |
| BIH | 3.118079 | 4 |
| BLR | 4.476406 | 6 |
| BLZ | 1.644636 | 1 |
| BMU | 3.836967 | 5 |
| BOL | 1.587078 | 1 |
| BRA | 2.841753 | 3 |
| BRB | 3.275399 | 4 |
| BRN | 2.306871 | 2 |
| BTN | 1.429757 | 1 |
| BWA | 1.587078 | 1 |
| CAF | 2.415885 | 2 |
| CAN | 3.498625 | 4 |
| CHE | 4.723332 | 6 |
| CHL | 2.059039 | 2 |
| CHN | 2.960411 | 3 |
| CIV | 1.744398 | 1 |
| CMR | 1.583546 | 1 |
| COD | 1.871174 | 1 |
| COG | 1.744398 | 1 |
| COL | 1.744398 | 1 |
| COM | 1.587078 | 1 |
| CPV | 1.644636 | 1 |
| CRI | 1.644636 | 1 |
| CUB | 4.104207 | 5 |
| CYM | 2.478641 | 3 |
| CYP | 2.736628 | 3 |
| CZE | 4.252275 | 6 |
| DEU | 4.972068 | 7 |
| DJI | 1.587078 | 1 |
| DMA | 2.531001 | 3 |
| DNK | 4.176214 | 5 |
| DOM | 1.529520 | 1 |
| DZA | 1.901719 | 2 |
| ECU | 1.744398 | 1 |
| EGY | 1.568355 | 1 |
| ERI | 1.686840 | 1 |
| ESP | 3.250794 | 4 |
| EST | 3.837872 | 5 |
| ETH | 1.399212 | 1 |
| FIN | 4.557665 | 6 |
| FJI | 2.116598 | 2 |
| FRA | 3.985941 | 5 |
| FSM | 1.744398 | 1 |
| GAB | 2.001481 | 2 |
| GBR | 3.613742 | 4 |
| GEO | 3.474924 | 4 |
| GHA | 1.671649 | 1 |
| GIN | 1.844161 | 1 |
| GMB | 1.529520 | 1 |
| GNB | 1.844161 | 1 |
| GNQ | 1.529520 | 1 |
| GRC | 3.895430 | 5 |
| GRD | 2.216360 | 2 |
| GRL | 3.928383 | 5 |
| GTM | 1.372199 | 1 |
| GUM | 2.778832 | 3 |
| GUY | 1.587078 | 1 |
| HND | 1.429757 | 1 |
| HRV | 3.747361 | 5 |
| HTI | 1.740866 | 1 |
| HUN | 3.747361 | 5 |
| IDN | 1.410515 | 1 |
| IMN | 2.693519 | 3 |
| IND | 2.031679 | 2 |
| IRL | 3.836967 | 5 |
| IRN | 1.587078 | 1 |
| IRQ | 1.429757 | 1 |
| ISL | 3.961335 | 5 |
| ISR | 2.827139 | 3 |
| ITA | 3.523231 | 4 |
| JAM | 1.801956 | 1 |
| JOR | 1.644636 | 1 |
| JPN | 4.856952 | 7 |
| KAZ | 3.102725 | 4 |
| KEN | 1.541341 | 1 |
| KGZ | 2.588559 | 3 |
| KHM | 1.583546 | 1 |
| KIR | 2.461946 | 2 |
| KNA | 2.149550 | 2 |
| KOR | 3.592448 | 4 |
| KWT | 2.537103 | 3 |
| LAO | 1.429757 | 1 |
| LBN | 2.067329 | 2 |
| LBR | 1.686840 | 1 |
| LBY | 2.373681 | 2 |
| LCA | 1.587078 | 1 |
| LIE | 4.156806 | 5 |
| LKA | 2.001481 | 2 |
| LSO | 2.331476 | 2 |
| LTU | 4.252275 | 6 |
| LUX | 4.290426 | 6 |
| LVA | 3.804919 | 5 |
| MAC | 2.763962 | 3 |
| MAR | 1.456770 | 1 |
| MCO | 7.133113 | 10 |
| MDA | 3.375162 | 4 |
| MDG | 1.686840 | 1 |
| MDV | 1.744398 | 1 |
| MEX | 1.928732 | 2 |
| MHL | 2.131951 | 2 |
| MKD | 2.903200 | 3 |
| MLI | 2.212828 | 2 |
| MLT | 3.995192 | 5 |
| MMR | 1.713853 | 1 |
| MNE | 3.060521 | 4 |
| MNG | 2.531001 | 3 |
| MNP | 2.306871 | 2 |
| MOZ | 1.786603 | 1 |
| MRT | 1.429757 | 1 |
| MUS | 2.531001 | 3 |
| MWI | 1.744398 | 1 |
| MYS | 1.686840 | 1 |
| NAM | 1.959277 | 2 |
| NCL | 2.373681 | 2 |
| NER | 2.001481 | 2 |
| NGA | 1.940034 | 2 |
| NIC | 1.372199 | 1 |
| NLD | 4.085703 | 5 |
| NOR | 4.738686 | 6 |
| NPL | 1.695130 | 1 |
| NRU | 2.304626 | 2 |
| NZL | 3.556183 | 4 |
| OMN | 1.801956 | 1 |
| PAK | 1.967047 | 2 |
| PAN | 1.801956 | 1 |
| PER | 1.587078 | 1 |
| PHL | 1.713853 | 1 |
| PLW | 2.174156 | 2 |
| PNG | 2.216360 | 2 |
| POL | 3.432720 | 4 |
| PRI | 2.993711 | 3 |
| PRK | 3.732007 | 5 |
| PRT | 3.680551 | 5 |
| PRY | 1.529520 | 1 |
| PSE | 1.314641 | 1 |
| PYF | 2.373681 | 2 |
| QAT | 1.943181 | 2 |
| RKS | 2.273918 | 2 |
| ROU | 3.532482 | 4 |
| RUS | 4.200080 | 5 |
| RWA | 1.456770 | 1 |
| SAU | 2.049788 | 2 |
| SDN | 1.372199 | 1 |
| SEN | 1.456770 | 1 |
| SGP | 2.771988 | 3 |
| SLB | 1.644636 | 1 |
| SLE | 2.028494 | 2 |
| SLV | 1.901719 | 2 |
| SMR | 3.928383 | 5 |
| SOM | 1.786603 | 1 |
| SRB | 3.590041 | 4 |
| SSD | 2.158802 | 2 |
| STP | 1.556533 | 1 |
| SUR | 2.001481 | 2 |
| SVK | 3.665198 | 5 |
| SVN | 3.507877 | 4 |
| SWE | 4.070349 | 5 |
| SWZ | 1.844161 | 1 |
| SYC | 2.001481 | 2 |
| SYR | 1.686840 | 1 |
| TCA | 1.777351 | 1 |
| TCD | 2.158802 | 2 |
| TGO | 1.587078 | 1 |
| THA | 2.086053 | 2 |
| TJK | 2.316122 | 2 |
| TKM | 2.745880 | 3 |
| TLS | 2.043848 | 2 |
| TON | 1.801956 | 1 |
| TTO | 2.803438 | 3 |
| TUN | 2.174156 | 2 |
| TUR | 2.273918 | 2 |
| TUV | 1.801956 | 1 |
| TZA | 1.556533 | 1 |
| UGA | 1.556533 | 1 |
| UKR | 4.004444 | 5 |
| URY | 3.118079 | 4 |
| USA | 3.665767 | 5 |
| UZB | 2.872655 | 3 |
| VCT | 2.116598 | 2 |
| VEN | 1.744398 | 1 |
| VIR | 4.252275 | 6 |
| VNM | 1.798425 | 1 |
| VUT | 1.644636 | 1 |
| WSM | 1.487315 | 1 |
| YEM | 1.556533 | 1 |
| ZAF | 2.331476 | 2 |
| ZMB | 1.901719 | 2 |
| ZWE | 1.801956 | 1 |

```
sp_data <- PreCOVID19_Risk[, c(1, 2)]
    map <- worldPlot(sp_data)
    map
```

```
## Scale on map varies by more than 10%, scale bar may be inaccurate
```

# 5 Post-COVID-19 - Regression Analysis

## 5.1 Post-COVID-19 Risk Evaluation Function

```
setwd("D:/VS/COVIDModel")
#-- covid19RiskEvaluation with Regression Function  ------------
covid19RiskEvaluation_Reg <- function(SI.date, df_c, df_d, df_r, BaseData_ss, si_source, CountryCodeList, iv_norm, iv_rev, dv, normalization, imp) {
    tryCatch({

        #
        si <- si_source[c("country_code", SI.date)]
        colnames(si) <- c("country_code", "stringency")
        #-----CovidCases Integration

        data_Cov_ss <- covid_data_day(SI.date, df_c, df_d, df_r)
        data_Cov_ss_g <- covid_data_day_growth(SI.date, df_c, df_d, df_r)

        colnames(CountryCodeList) <- c("country", "country_code")
        data_Cov_ss1 <- merge(data_Cov_ss, CountryCodeList, by = 'country', all.x = T)

        #data_Cov_ss1 <- merge(data_Cov_ss, CountryCodeList, by = 'country', all.x = T)

        data_Cov_ss2 <- data.frame(country_code = data_Cov_ss1$country_code, deaths = data_Cov_ss1$deaths,
                           confirmed = data_Cov_ss1$confirmed, active = data_Cov_ss1$active,
                           deathsGrowth = data_Cov_ss_g$deathsGrowth,
                           confirmedGrowth = data_Cov_ss_g$confirmedGrowth,
                           recoveredGrowth = data_Cov_ss_g$recoveredGrowth)

        #----- Data Integration
        data_m_si <- merge(si, BaseData_ss, by = "country_code", all.x = T)
        data_m <- merge(data_m_si, data_Cov_ss2, by = "country_code", all.x = T)

        data_m$susceptibles = (data_m$Pop_2018 - data_m$confirmed) * 100 / data_m$Pop_2018
        data_m$deaths = (data_m$deaths) / data_m$Pop_2018 * 1000000
        data_m$confirmed = (data_m$confirmed) / data_m$Pop_2018 * 1000000
        data_m$active = (data_m$active) / data_m$Pop_2018 * 1000000

        #################
        if (normalization == "BoxCox") {
            #---------- Weight Model Preparation 
            data_num <- sapply(data_m[, 2:ncol(data_m)], as.numeric)
            data_num <- cbind(data_m[, 1:2], data_num)
            data_a <- data_num[apply(data_num[, 2:ncol(data_num)] > 0, 1, all),]
            data_a <- na.omit(data_a)
            data_a <- data_a[, -3]

            #--Split Tab for normalization

            data_sp <- data_a[, 2:ncol(data_a)]
            data_sp <- sapply(data_sp, as.numeric)

            data_normal <- boxcox(data_sp)
            data_normal <- as.data.frame(data_normal$x.t)
            data_normal <- cbind(data_a["country_code"], data_normal)

        }
        ############################

        ##########################
        if (normalization == "Scaling") {
            data_num <- sapply(data_m[, 2:ncol(data_m)], as.numeric)
      
            data_normal <- scale(data_num)
 
            data_normal <- cbind(data_m["country_code"], data_normal)
        }
        ##########################

        #-- Weights and weighted data Preparation

        #imp <- imp
        #imp@lmg

        #------- Binning with Weights

        for (i in iv_norm) {
            #if (colnames(data_normal[i]) == "confirmed") {
            data_normal[i] <- cut(as.numeric(unlist(data_normal[i])), 10, labels = c(1, 2, 3, 4, 5, 6, 7, 8, 9, 10))
            data_normal[i] <- as.numeric(unlist(data_normal[i])) * imp[i]
        }

        for (i in iv_rev) {
            #if (colnames(data_normal[i]) == "confirmed") {
            data_normal[i] <- cut(as.numeric(unlist(data_normal[i])), 10, labels = c(10, 9, 8, 7, 6, 5, 4, 3, 2, 1))
            data_normal[i] <- as.numeric(unlist(data_normal[i])) * imp[i]
        }

        # --Risk Aggregation
        iv = append(iv_norm, iv_rev)
        RiskVariables <- append(c("country_code"), iv)
        data_Risk <- data_normal[RiskVariables]
        data_Risk <- mutate(data_Risk, RiskValue = rowSums(data_Risk[, -1], na.rm = TRUE))

        data_Risk <- transform(data_Risk, RiskValue = as.numeric(unlist(RiskValue)))
        #data_Risk_ss <- data_Risk[, c(1, 2, 3, 4, 5, 6, 7, 8, 9, 10, 16, 17)]

        data_Risk$RiskRank <- cut(data_Risk[, "RiskValue"], 10, labels = c(1, 2, 3, 4, 5, 6, 7, 8, 9, 10))

        Risk <- data_Risk[c("country_code", "RiskValue", "RiskRank")]
        colnames(Risk) <- c("country_code", str_c("RiskValue_", SI.date), str_c("RiskRank_", SI.date))

        return(Risk)
    },
    error = function(err) {
        return("Please check your data")
    })
}
```

## 5.2 Post-COVID-19 Static Model

### 5.2.1 Static Weights Extraction

```
setwd("D:/VS/COVIDModel")
#-- Regression Model Results -------------
#
df_ModelRsquare = NULL
df_ModelWeights = NULL
df_ModelSummary = NULL
df_pValue = NULL
df_vif = NULL

    SI.date = SI.date
    BaseData_ss <- BaseData[, c(1, 3:length(BaseData))]
    # COVID-19 Data Integration
    df_c = read.csv("d:/VS/COVIDModel/Data/COVID-19-master/csse_covid_19_data/csse_covid_19_time_series/time_series_covid19_confirmed_global.csv",
                         stringsAsFactors = FALSE)
    df_d = read.csv("d:/VS/COVIDModel/Data/COVID-19-master/csse_covid_19_data/csse_covid_19_time_series/time_series_covid19_deaths_global.csv",
                         stringsAsFactors = FALSE)
    df_r = read.csv("d:/VS/COVIDModel/Data/COVID-19-master/csse_covid_19_data/csse_covid_19_time_series/time_series_covid19_recovered_global.csv",
                         stringsAsFactors = FALSE)
    dv = "deaths"

    normalization = "BoxCox"

    #### 

    iv_norm <- c("DALYs", "Pop_2018", "A65abp_2018", "PopDen_Avg", "Physician", "Nurses", "HospitalBeds",  
            "GDP_PC", "Expenditure" , "stringency")

    
    regModel <- covid19_RegModel(SI.date, df_c, df_d, df_r, BaseData_ss, si_source, CountryCodeList, iv_norm, iv_rev, dv, normalization)
```

```
## `summarise()` ungrouping output (override with `.groups` argument)
## `summarise()` ungrouping output (override with `.groups` argument)
## `summarise()` ungrouping output (override with `.groups` argument)
## `summarise()` ungrouping output (override with `.groups` argument)
## `summarise()` ungrouping output (override with `.groups` argument)
## `summarise()` ungrouping output (override with `.groups` argument)
## `summarise()` ungrouping output (override with `.groups` argument)
## `summarise()` ungrouping output (override with `.groups` argument)
## `summarise()` ungrouping output (override with `.groups` argument)
```

```
    impPost <- regModel[[2]]$lmg
    

    weights <- data.frame(t(data.frame(regModel[[2]]$lmg)))
    c <- colnames(weights)
    c1 <- NULL
    for (i in 1:length(c)) {
        c2 <- str_c("wt_", c[i])
        c1 <- append(c1, c2)
    }
    colnames(weights) <- c1

    Date <- data.frame(Date = SI.date)
    weights <- cbind(Date, weights)
    df_ModelWeights <- rbind(df_ModelWeights, weights)

    
    p.value <- data.frame(t(data.frame(regModel[[1]]$coefficients[, 4])))
    c <- colnames(p.value)
    c1 <- c("pv_Intercept")
    for (i in 2:length(c)) {
        c2 <- str_c("pv_", c[i])
        c1 <- append(c1, c2)
    }
    colnames(p.value) <- c1
    df_pValue <- rbind(df_pValue, p.value)
    df_ModelSummary <- cbind(df_ModelWeights, df_pValue)

    vif.value <- data.frame(t(data.frame(regModel[[3]])))
    d <- colnames(vif.value)
    #c1 <- c("pv_Intercept")
    d1<- NULL
    for (i in 1:length(d)) {
        d2 <- str_c("vif_", d[i])
        d1 <- append(d1, d2)
    }
    colnames(vif.value) <- d1
    df_vif <- rbind(df_vif, vif.value)
    df_ModelSummary <- cbind(df_ModelSummary, df_vif)

    rsquare <- data.frame(Date = SI.date, RSquare = regModel[[1]]$r.squared)
    df_ModelRsquare <- rbind(df_ModelRsquare, rsquare)
    df_ModelSummary <- cbind(df_ModelSummary, df_ModelRsquare[2])

    print(str_c("Data prepared for ", SI.date, " R-Square = ", regModel[[1]]$r.squared))
```

```
## [1] "Data prepared for 2020-05-13 R-Square = 0.685140962280837"
```

```
# The Outcome of Regression Model - Weights and P-Values
rownames(df_ModelSummary) <-c()
kable(df_ModelSummary) %>%
    kable_styling("striped", full_width = F, fixed_thead = T) %>%
    row_spec(0) %>%
    scroll_box(width = "910px", height = "440px")
```

| Date | wt\_DALYs | wt\_Pop\_2018 | wt\_A65abp\_2018 | wt\_PopDen\_Avg | wt\_Physician | wt\_Nurses | wt\_HospitalBeds | wt\_GDP\_PC | wt\_Expenditure | wt\_stringency | pv\_Intercept | pv\_DALYs | pv\_Pop\_2018 | pv\_A65abp\_2018 | pv\_PopDen\_Avg | pv\_Physician | pv\_Nurses | pv\_HospitalBeds | pv\_GDP\_PC | pv\_Expenditure | pv\_stringency | vif\_DALYs | vif\_Pop\_2018 | vif\_A65abp\_2018 | vif\_PopDen\_Avg | vif\_Physician | vif\_Nurses | vif\_HospitalBeds | vif\_GDP\_PC | vif\_Expenditure | vif\_stringency | RSquare |
| --- | --- | --- | --- | --- | --- | --- | --- | --- | --- | --- | --- | --- | --- | --- | --- | --- | --- | --- | --- | --- | --- | --- | --- | --- | --- | --- | --- | --- | --- | --- | --- | --- |
| 2020-05-13 | 0.0553956 | 0.0180688 | 0.1897825 | 0.0431309 | 0.1394969 | 0.159799 | 0.1014566 | 0.2204155 | 0.0400847 | 0.0323696 | 0.8707628 | 0.8328308 | 0.9282236 | 0.0082958 | 0.4604002 | 0.6528591 | 0.1599155 | 0.0992787 | 0.062661 | 0.832533 | 0.0818878 | 3.135882 | 1.369651 | 3.745179 | 1.524102 | 6.112762 | 6.440584 | 5.897403 | 8.048334 | 1.699711 | 1.285996 | 0.685141 |

```
weight <- data.frame(impPost)
colnames(weight) <- c("weight")
kable(weight) %>%
     kable_styling("striped", full_width = F, fixed_thead = T) %>%
     row_spec(0) %>%
     scroll_box(width = "910px", height = "440px")
```

|  | weight |
| --- | --- |
| DALYs | 0.0553956 |
| Pop\_2018 | 0.0180688 |
| A65abp\_2018 | 0.1897825 |
| PopDen\_Avg | 0.0431309 |
| Physician | 0.1394969 |
| Nurses | 0.1597990 |
| HospitalBeds | 0.1014566 |
| GDP\_PC | 0.2204155 |
| Expenditure | 0.0400847 |
| stringency | 0.0323696 |

### 5.2.2 Static Risk Evaluation

```
    setwd("D:/VS/COVIDModel")
    PostCOVID19_StaticRisk <- covid19RiskEvaluation_Reg(SI.date = SI.date,
    df_c = read.csv("d:/VS/COVIDModel/Data/COVID-19-master/csse_covid_19_data/csse_covid_19_time_series/time_series_covid19_confirmed_global.csv",
                         stringsAsFactors = FALSE),
    df_d = read.csv("d:/VS/COVIDModel/Data/COVID-19-master/csse_covid_19_data/csse_covid_19_time_series/time_series_covid19_deaths_global.csv",
                         stringsAsFactors = FALSE),
    df_r = read.csv("d:/VS/COVIDModel/Data/COVID-19-master/csse_covid_19_data/csse_covid_19_time_series/time_series_covid19_recovered_global.csv",
                         stringsAsFactors = FALSE),
    BaseData_ss <- BaseData[, c(1, 3:13)],
    si_source = si_source,

    #country code path 
    CountryCodeList <- read.csv("./data/country_JHU.csv", stringsAsFactors = FALSE, check.names = FALSE, fileEncoding = "UTF-8-BOM"),
    iv_norm = c("DALYs", "Pop_2018", "A65abp_2018", "PopDen_Avg", "Physician", "Nurses", "HospitalBeds"),
    iv_rev = c("GDP_PC", "Expenditure", "stringency"),
    dv = "deaths",
    normalization = "Scaling",
     imp = impPost)
```

```
## `summarise()` ungrouping output (override with `.groups` argument)
## `summarise()` ungrouping output (override with `.groups` argument)
## `summarise()` ungrouping output (override with `.groups` argument)
## `summarise()` ungrouping output (override with `.groups` argument)
## `summarise()` ungrouping output (override with `.groups` argument)
## `summarise()` ungrouping output (override with `.groups` argument)
## `summarise()` ungrouping output (override with `.groups` argument)
## `summarise()` ungrouping output (override with `.groups` argument)
## `summarise()` ungrouping output (override with `.groups` argument)
```

### 5.2.3 Static Risk Results

```
kable(PostCOVID19_StaticRisk) %>%
     kable_styling("striped", full_width = F, fixed_thead = T) %>%
     row_spec(0) %>%
     scroll_box(width = "910px", height = "440px")
```

| country\_code | RiskValue\_2020-05-13 | RiskRank\_2020-05-13 |
| --- | --- | --- |
| ABW | 2.8859809 | 5 |
| AFG | 1.7963368 | 3 |
| AGO | 1.6128864 | 3 |
| ALB | 2.5496437 | 4 |
| AND | 4.0222982 | 7 |
| ARE | 2.4750328 | 4 |
| ARG | 2.8396077 | 5 |
| AUS | 4.8384578 | 8 |
| AUT | 5.4446295 | 9 |
| AZE | 2.8858823 | 5 |
| BDI | 1.5388893 | 3 |
| BEL | 5.3667184 | 9 |
| BEN | 1.3571472 | 3 |
| BFA | 1.7036582 | 3 |
| BGD | 1.6175125 | 3 |
| BGR | 4.0739798 | 7 |
| BHR | 2.1343612 | 4 |
| BIH | 3.3492808 | 6 |
| BLZ | 1.6174680 | 3 |
| BMU | 5.3734378 | 9 |
| BOL | 1.9844092 | 4 |
| BRA | 3.0362485 | 5 |
| BRB | 3.7552542 | 6 |
| BRN | 2.7385648 | 5 |
| BWA | 1.7775636 | 3 |
| CAN | 4.2945333 | 7 |
| CHE | 6.3620374 | 10 |
| CHL | 2.4190599 | 4 |
| CHN | 2.8559932 | 5 |
| CIV | 1.5497362 | 3 |
| CMR | 1.6375408 | 3 |
| COD | 1.4988046 | 3 |
| COG | 1.5310581 | 3 |
| COL | 1.8943980 | 3 |
| CPV | 1.8383842 | 3 |
| CRI | 2.2051838 | 4 |
| CUB | 4.4514496 | 7 |
| CYP | 3.3944783 | 6 |
| CZE | 4.4852861 | 8 |
| DEU | 5.8770840 | 10 |
| DJI | 1.7105254 | 3 |
| DMA | 2.8147887 | 5 |
| DNK | 5.4846140 | 9 |
| DOM | 2.0025646 | 4 |
| DZA | 2.0536362 | 4 |
| ECU | 1.9066627 | 3 |
| EGY | 1.9907173 | 4 |
| ESP | 4.0709737 | 7 |
| EST | 4.3017770 | 7 |
| ETH | 1.4944657 | 3 |
| FIN | 5.7746423 | 10 |
| FRA | 5.1046942 | 9 |
| GAB | 1.8119815 | 3 |
| GBR | 4.3858844 | 7 |
| GHA | 1.5513984 | 3 |
| GMB | 1.3772521 | 3 |
| GRC | 4.1915591 | 7 |
| GRL | 4.6543304 | 8 |
| GTM | 1.4238739 | 3 |
| GUM | 3.4853788 | 6 |
| GUY | 1.8506780 | 3 |
| HND | 1.4702560 | 3 |
| HRV | 4.3900985 | 7 |
| HUN | 4.2144279 | 7 |
| IDN | 1.6017417 | 3 |
| IND | 1.8448188 | 3 |
| IRL | 4.9682671 | 8 |
| IRN | 1.6970976 | 3 |
| IRQ | 1.4199704 | 3 |
| ISL | 5.5988868 | 9 |
| ISR | 3.7174716 | 6 |
| ITA | 4.6108775 | 8 |
| JAM | 1.9641026 | 4 |
| JOR | 1.9368688 | 4 |
| JPN | 5.7943621 | 10 |
| KAZ | 3.1635168 | 5 |
| KEN | 1.5391338 | 3 |
| KGZ | 2.5514930 | 4 |
| KOR | 4.2250656 | 7 |
| KWT | 2.8764380 | 5 |
| LAO | 1.5810471 | 3 |
| LBN | 2.5007007 | 4 |
| LBR | 1.4619710 | 3 |
| LBY | 2.6164337 | 5 |
| LKA | 2.3334680 | 4 |
| LUX | 5.8576210 | 10 |
| MAR | 1.8016958 | 3 |
| MDA | 3.0661812 | 5 |
| MDG | 1.5004330 | 3 |
| MEX | 2.0802692 | 4 |
| MLI | 1.9131009 | 3 |
| MMR | 1.7019819 | 3 |
| MNG | 2.6925926 | 5 |
| MOZ | 1.4632694 | 3 |
| MRT | 1.3573917 | 3 |
| MUS | 2.6547925 | 5 |
| MWI | 1.4804475 | 3 |
| MYS | 2.1481357 | 4 |
| NAM | 1.7263922 | 3 |
| NER | 1.6234888 | 3 |
| NGA | 1.6015923 | 3 |
| NIC | 1.4495335 | 3 |
| NLD | 5.2422358 | 9 |
| NOR | 6.1681345 | 10 |
| NZL | 4.4094583 | 7 |
| OMN | 2.1532569 | 4 |
| PAK | 1.8479006 | 3 |
| PAN | 2.1398839 | 4 |
| PER | 1.9241430 | 3 |
| PHL | 1.7143717 | 3 |
| PNG | 1.6879102 | 3 |
| POL | 3.8746269 | 7 |
| PRI | 3.6726861 | 6 |
| PRT | 4.2560751 | 7 |
| PRY | 1.7702120 | 3 |
| PSE | 1.4472506 | 3 |
| QAT | 2.9544216 | 5 |
| ROU | 3.9854180 | 7 |
| RUS | 4.1258231 | 7 |
| RWA | 1.5065287 | 3 |
| SAU | 2.3130559 | 4 |
| SDN | 1.2463561 | 2 |
| SEN | 1.4050721 | 3 |
| SGP | 3.7300731 | 6 |
| SLE | 1.7174657 | 3 |
| SLV | 2.0254912 | 4 |
| SMR | 5.3587479 | 9 |
| SOM | 1.4525081 | 3 |
| SRB | 3.7877319 | 6 |
| SSD | 1.6542299 | 3 |
| SUR | 2.3620118 | 4 |
| SVK | 3.8821439 | 7 |
| SVN | 4.3894505 | 7 |
| SWE | 5.3184367 | 9 |
| SWZ | 1.8959504 | 3 |
| SYC | 2.2931501 | 4 |
| SYR | 1.6187664 | 3 |
| TCD | 1.6758382 | 3 |
| THA | 2.4348012 | 4 |
| TTO | 2.9546347 | 5 |
| TUN | 2.2164608 | 4 |
| TUR | 2.2588490 | 4 |
| TWN | 0.0323696 | 1 |
| TZA | 1.4635139 | 3 |
| UGA | 1.3880134 | 3 |
| UKR | 4.0701343 | 7 |
| URY | 3.8562124 | 7 |
| USA | 4.5174007 | 8 |
| UZB | 3.0664218 | 5 |
| VEN | 2.1594477 | 4 |
| VNM | 2.0411460 | 4 |
| YEM | 1.4958835 | 3 |
| ZAF | 2.0957563 | 4 |
| ZMB | 1.6035123 | 3 |
| ZWE | 1.6112275 | 3 |

```
sp_data <- PostCOVID19_StaticRisk[, c(1, 2)]
map <- worldPlot(sp_data)
map
```

```
## Scale on map varies by more than 10%, scale bar may be inaccurate
```

---

## 5.3 Post-COVID-19 Dynamic Model

### 5.3.1 Dynamic Weights Extraction

```
setwd("D:/VS/COVIDModel")
#-- Regression Model Results -------------
#
df_ModelRsquare = NULL
df_ModelWeights = NULL
df_ModelSummary = NULL
df_pValue = NULL
df_vif = NULL

    SI.date = SI.date
    BaseData_ss <- BaseData[, c(1, 3:length(BaseData))]
    # COVID-19 Data Integration
    df_c = read.csv("d:/VS/COVIDModel/Data/COVID-19-master/csse_covid_19_data/csse_covid_19_time_series/time_series_covid19_confirmed_global.csv",
                         stringsAsFactors = FALSE)
    df_d = read.csv("d:/VS/COVIDModel/Data/COVID-19-master/csse_covid_19_data/csse_covid_19_time_series/time_series_covid19_deaths_global.csv",
                         stringsAsFactors = FALSE)
    df_r = read.csv("d:/VS/COVIDModel/Data/COVID-19-master/csse_covid_19_data/csse_covid_19_time_series/time_series_covid19_recovered_global.csv",
                         stringsAsFactors = FALSE)
    dv = "deaths"

    normalization = "BoxCox"

    #### 

    iv_norm <- c("DALYs", "Pop_2018", "A65abp_2018", "PopDen_Avg", "Physician", "Nurses", "HospitalBeds", "susceptibles" , "active" , "confirmedGrowth" , "deathsGrowth", 
            "GDP_PC", "Expenditure" , "stringency" , "recoveredGrowth" )

    
    regModel <- covid19_RegModel(SI.date, df_c, df_d, df_r, BaseData_ss, si_source, CountryCodeList, iv_norm, iv_rev, dv, normalization)
```

```
## `summarise()` ungrouping output (override with `.groups` argument)
## `summarise()` ungrouping output (override with `.groups` argument)
## `summarise()` ungrouping output (override with `.groups` argument)
## `summarise()` ungrouping output (override with `.groups` argument)
## `summarise()` ungrouping output (override with `.groups` argument)
## `summarise()` ungrouping output (override with `.groups` argument)
## `summarise()` ungrouping output (override with `.groups` argument)
## `summarise()` ungrouping output (override with `.groups` argument)
## `summarise()` ungrouping output (override with `.groups` argument)
```

```
    impPost <- regModel[[2]]$lmg
    

    weights <- data.frame(t(data.frame(regModel[[2]]$lmg)))
    c <- colnames(weights)
    c1 <- NULL
    for (i in 1:length(c)) {
        c2 <- str_c("wt_", c[i])
        c1 <- append(c1, c2)
    }
    colnames(weights) <- c1

    Date <- data.frame(Date = SI.date)
    weights <- cbind(Date, weights)
    df_ModelWeights <- rbind(df_ModelWeights, weights)

    
    p.value <- data.frame(t(data.frame(regModel[[1]]$coefficients[, 4])))
    c <- colnames(p.value)
    c1 <- c("pv_Intercept")
    for (i in 2:length(c)) {
        c2 <- str_c("pv_", c[i])
        c1 <- append(c1, c2)
    }
    colnames(p.value) <- c1
    df_pValue <- rbind(df_pValue, p.value)
    df_ModelSummary <- cbind(df_ModelWeights, df_pValue)

    vif.value <- data.frame(t(data.frame(regModel[[3]])))
    d <- colnames(vif.value)
    #c1 <- c("pv_Intercept")
    d1<- NULL
    for (i in 1:length(d)) {
        d2 <- str_c("vif_", d[i])
        d1 <- append(d1, d2)
    }
    colnames(vif.value) <- d1
    df_vif <- rbind(df_vif, vif.value)
    df_ModelSummary <- cbind(df_ModelSummary, df_vif)

    rsquare <- data.frame(Date = SI.date, RSquare = regModel[[1]]$r.squared)
    df_ModelRsquare <- rbind(df_ModelRsquare, rsquare)
    df_ModelSummary <- cbind(df_ModelSummary, df_ModelRsquare[2])

    print(str_c("Data prepared for ", SI.date, " R-Square = ", regModel[[1]]$r.squared))
```

```
## [1] "Data prepared for 2020-05-13 R-Square = 0.884346780535134"
```

```
# The Outcome of Regression Model - Weights and P-Values
rownames(df_ModelSummary) <-c()
kable(df_ModelSummary) %>%
    kable_styling("striped", full_width = F, fixed_thead = T) %>%
    row_spec(0) %>%
    scroll_box(width = "910px", height = "440px")
```

| Date | wt\_DALYs | wt\_Pop\_2018 | wt\_A65abp\_2018 | wt\_PopDen\_Avg | wt\_Physician | wt\_Nurses | wt\_HospitalBeds | wt\_susceptibles | wt\_active | wt\_confirmedGrowth | wt\_deathsGrowth | wt\_GDP\_PC | wt\_Expenditure | wt\_stringency | wt\_recoveredGrowth | pv\_Intercept | pv\_DALYs | pv\_Pop\_2018 | pv\_A65abp\_2018 | pv\_PopDen\_Avg | pv\_Physician | pv\_Nurses | pv\_HospitalBeds | pv\_susceptibles | pv\_active | pv\_confirmedGrowth | pv\_deathsGrowth | pv\_GDP\_PC | pv\_Expenditure | pv\_stringency | pv\_recoveredGrowth | vif\_DALYs | vif\_Pop\_2018 | vif\_A65abp\_2018 | vif\_PopDen\_Avg | vif\_Physician | vif\_Nurses | vif\_HospitalBeds | vif\_susceptibles | vif\_active | vif\_confirmedGrowth | vif\_deathsGrowth | vif\_GDP\_PC | vif\_Expenditure | vif\_stringency | vif\_recoveredGrowth | RSquare |
| --- | --- | --- | --- | --- | --- | --- | --- | --- | --- | --- | --- | --- | --- | --- | --- | --- | --- | --- | --- | --- | --- | --- | --- | --- | --- | --- | --- | --- | --- | --- | --- | --- | --- | --- | --- | --- | --- | --- | --- | --- | --- | --- | --- | --- | --- | --- | --- |
| 2020-05-13 | 0.0263156 | 0.0099665 | 0.1038412 | 0.0232691 | 0.0711147 | 0.0803109 | 0.0520602 | 0.1491394 | 0.1989307 | 0.0418155 | 0.1096596 | 0.0937475 | 0.0220299 | 0.0135328 | 0.0042662 | 0.1015172 | 0.6255222 | 0.4589768 | 0.0007561 | 0.6044021 | 0.507399 | 0.0366399 | 0.1497271 | 0.0146218 | 1.37e-05 | 0.6223965 | 0.0019193 | 0.4896054 | 0.6197291 | 0.1123584 | 0.6842231 | 3.244279 | 1.701685 | 4.475173 | 1.666843 | 6.790883 | 6.789272 | 6.280662 | 3.473249 | 4.011424 | 3.77267 | 2.846386 | 10.60096 | 1.791639 | 1.427008 | 1.584557 | 0.8843468 |

```
weight <- data.frame(impPost)
colnames(weight) <- c("weight")
kable(weight) %>%
     kable_styling("striped", full_width = F, fixed_thead = T) %>%
     row_spec(0) %>%
     scroll_box(width = "910px", height = "440px")
```

|  | weight |
| --- | --- |
| DALYs | 0.0263156 |
| Pop\_2018 | 0.0099665 |
| A65abp\_2018 | 0.1038412 |
| PopDen\_Avg | 0.0232691 |
| Physician | 0.0711147 |
| Nurses | 0.0803109 |
| HospitalBeds | 0.0520602 |
| susceptibles | 0.1491394 |
| active | 0.1989307 |
| confirmedGrowth | 0.0418155 |
| deathsGrowth | 0.1096596 |
| GDP\_PC | 0.0937475 |
| Expenditure | 0.0220299 |
| stringency | 0.0135328 |
| recoveredGrowth | 0.0042662 |

### 5.3.2 Dynamic Risk Evaluation

```
    setwd("D:/VS/COVIDModel")
    PostCOVID19_DynamicRisk <- covid19RiskEvaluation_Reg(SI.date = SI.date,
    df_c = read.csv("d:/VS/COVIDModel/Data/COVID-19-master/csse_covid_19_data/csse_covid_19_time_series/time_series_covid19_confirmed_global.csv",
                         stringsAsFactors = FALSE),
    df_d = read.csv("d:/VS/COVIDModel/Data/COVID-19-master/csse_covid_19_data/csse_covid_19_time_series/time_series_covid19_deaths_global.csv",
                         stringsAsFactors = FALSE),
    df_r = read.csv("d:/VS/COVIDModel/Data/COVID-19-master/csse_covid_19_data/csse_covid_19_time_series/time_series_covid19_recovered_global.csv",
                         stringsAsFactors = FALSE),
    BaseData_ss <- BaseData[, c(1, 3:13)],
    si_source = si_source,

    #country code path 
    CountryCodeList <- read.csv("./data/country_JHU.csv", stringsAsFactors = FALSE, check.names = FALSE, fileEncoding = "UTF-8-BOM"),
    iv_norm = c("DALYs", "Pop_2018", "A65abp_2018", "PopDen_Avg", "Physician", "Nurses", "HospitalBeds", "susceptibles", "active", "confirmedGrowth", "deathsGrowth"),
    iv_rev = c("GDP_PC", "Expenditure", "stringency", "recoveredGrowth"),
    dv = "deaths",
    normalization = "Scaling",
     imp = impPost)
```

```
## `summarise()` ungrouping output (override with `.groups` argument)
## `summarise()` ungrouping output (override with `.groups` argument)
## `summarise()` ungrouping output (override with `.groups` argument)
## `summarise()` ungrouping output (override with `.groups` argument)
## `summarise()` ungrouping output (override with `.groups` argument)
## `summarise()` ungrouping output (override with `.groups` argument)
## `summarise()` ungrouping output (override with `.groups` argument)
## `summarise()` ungrouping output (override with `.groups` argument)
## `summarise()` ungrouping output (override with `.groups` argument)
```

### 5.3.3 Dynamic Risk Results

```
kable(PostCOVID19_DynamicRisk) %>%
     kable_styling("striped", full_width = F, fixed_thead = T) %>%
     row_spec(0) %>%
     scroll_box(width = "910px", height = "440px")
```

| country\_code | RiskValue\_2020-05-13 | RiskRank\_2020-05-13 |
| --- | --- | --- |
| ABW | 3.2963017 | 7 |
| AFG | 2.7833100 | 6 |
| AGO | 2.6562681 | 6 |
| ALB | 3.1619273 | 7 |
| AND | 3.3142014 | 7 |
| ARE | 3.0827662 | 6 |
| ARG | 3.3487784 | 7 |
| AUS | 4.2568936 | 9 |
| AUT | 4.5781456 | 9 |
| AZE | 3.4233777 | 7 |
| BDI | 2.6127764 | 5 |
| BEL | 4.6348133 | 10 |
| BEN | 2.5178926 | 5 |
| BFA | 2.6827369 | 6 |
| BGD | 2.8218133 | 6 |
| BGR | 3.9599068 | 8 |
| BHR | 3.2033277 | 7 |
| BIH | 3.5734026 | 7 |
| BLZ | 2.6672086 | 6 |
| BMU | 4.4794975 | 9 |
| BOL | 2.9944745 | 6 |
| BRA | 3.5423684 | 7 |
| BRB | 3.7682756 | 8 |
| BRN | 3.1967813 | 7 |
| BWA | 2.7455335 | 6 |
| CAN | 3.8567695 | 8 |
| CHE | 4.8264186 | 10 |
| CHL | 3.1043517 | 6 |
| CHN | 3.3170087 | 7 |
| CIV | 2.8261558 | 6 |
| CMR | 2.8127789 | 6 |
| COD | 2.8518813 | 6 |
| COG | 2.6055155 | 5 |
| COL | 2.8589035 | 6 |
| CPV | 2.7982316 | 6 |
| CRI | 3.1849113 | 7 |
| CUB | 4.1332012 | 9 |
| CYP | 3.6608393 | 8 |
| CZE | 4.1424512 | 9 |
| DEU | 4.6493585 | 10 |
| DJI | 2.6943590 | 6 |
| DMA | 3.2776110 | 7 |
| DNK | 4.5809755 | 9 |
| DOM | 2.8654367 | 6 |
| DZA | 2.8854663 | 6 |
| ECU | 3.0190653 | 6 |
| EGY | 2.8546827 | 6 |
| ESP | 3.8121682 | 8 |
| EST | 4.0329448 | 8 |
| ETH | 2.5794634 | 5 |
| FIN | 4.7476336 | 10 |
| FRA | 4.4847035 | 9 |
| GAB | 2.8792594 | 6 |
| GBR | 4.3035273 | 9 |
| GHA | 2.7789493 | 6 |
| GMB | 2.5701943 | 5 |
| GRC | 4.0053701 | 8 |
| GRL | 4.1498904 | 9 |
| GTM | 2.7593466 | 6 |
| GUM | 1.7135376 | 4 |
| GUY | 2.7965992 | 6 |
| HND | 2.6336784 | 5 |
| HRV | 4.1003483 | 8 |
| HUN | 4.0038191 | 8 |
| IDN | 2.6981490 | 6 |
| IND | 2.9371256 | 6 |
| IRL | 3.9816409 | 8 |
| IRN | 2.7034596 | 6 |
| IRQ | 2.5966856 | 5 |
| ISL | 4.3067317 | 9 |
| ISR | 3.7079598 | 8 |
| ITA | 4.2571098 | 9 |
| JAM | 2.8536918 | 6 |
| JOR | 2.8291204 | 6 |
| JPN | 4.7926950 | 10 |
| KAZ | 3.4435878 | 7 |
| KEN | 2.8437469 | 6 |
| KGZ | 3.1451730 | 7 |
| KOR | 3.9845784 | 8 |
| KWT | 3.4724244 | 7 |
| LAO | 2.6402280 | 6 |
| LBN | 3.1356121 | 7 |
| LBR | 2.5712543 | 5 |
| LBY | 3.1553260 | 7 |
| LKA | 3.0482679 | 6 |
| LUX | 4.2264427 | 9 |
| MAR | 2.7642918 | 6 |
| MDA | 3.4546853 | 7 |
| MDG | 2.7084457 | 6 |
| MEX | 3.0628962 | 6 |
| MLI | 2.8935551 | 6 |
| MMR | 2.7087854 | 6 |
| MNG | 3.2089020 | 7 |
| MOZ | 2.5632659 | 5 |
| MRT | 3.8928199 | 8 |
| MUS | 3.1985902 | 7 |
| MWI | 2.6688526 | 6 |
| MYS | 2.9396719 | 6 |
| NAM | 2.7133070 | 6 |
| NER | 2.6386770 | 6 |
| NGA | 2.6435885 | 6 |
| NIC | 2.5833003 | 5 |
| NLD | 4.5585749 | 9 |
| NOR | 5.1032679 | 10 |
| NZL | 4.0535040 | 8 |
| OMN | 2.9564114 | 6 |
| PAK | 2.7806303 | 6 |
| PAN | 2.7774545 | 6 |
| PER | 3.0276046 | 6 |
| PHL | 2.7107745 | 6 |
| PNG | 2.6838913 | 6 |
| POL | 3.8352633 | 8 |
| PRI | 1.8600401 | 4 |
| PRT | 4.0858367 | 8 |
| PRY | 2.8599187 | 6 |
| PSE | 2.5660447 | 5 |
| QAT | 3.5459230 | 7 |
| ROU | 3.8878946 | 8 |
| RUS | 4.0081912 | 8 |
| RWA | 2.5994224 | 5 |
| SAU | 3.0409886 | 6 |
| SDN | 2.7724778 | 6 |
| SEN | 2.6988374 | 6 |
| SGP | 3.7657178 | 8 |
| SLE | 3.3759895 | 7 |
| SLV | 2.8789155 | 6 |
| SMR | 4.9924114 | 10 |
| SOM | 2.5953451 | 5 |
| SRB | 3.8089159 | 8 |
| SSD | 2.7017726 | 6 |
| SUR | 3.0366161 | 6 |
| SVK | 3.8229787 | 8 |
| SVN | 4.0611657 | 8 |
| SWE | 4.5617159 | 9 |
| SWZ | 2.8110182 | 6 |
| SYC | 2.9959217 | 6 |
| SYR | 2.6634864 | 6 |
| TCD | 2.7055690 | 6 |
| THA | 3.0958482 | 6 |
| TTO | 3.3436449 | 7 |
| TUN | 2.9757855 | 6 |
| TUR | 3.0063019 | 6 |
| TWN | 0.1692741 | 1 |
| TZA | 2.5749171 | 5 |
| UGA | 2.5799307 | 5 |
| UKR | 3.9324549 | 8 |
| URY | 3.8035443 | 8 |
| USA | 4.1771130 | 9 |
| UZB | 3.5093011 | 7 |
| VEN | 2.9274149 | 6 |
| VNM | 2.8941565 | 6 |
| YEM | 2.9592443 | 6 |
| ZAF | 3.0617680 | 6 |
| ZMB | 2.6410782 | 6 |
| ZWE | 2.6581078 | 6 |

```
sp_data <- PostCOVID19_DynamicRisk[, c(1, 2)]
map <- worldPlot(sp_data)
map
```

```
## Scale on map varies by more than 10%, scale bar may be inaccurate
```

---

# 6 Difference of Pre COVID and Post COVID Dynamic Risk

```
RiskTable <- merge(PreCOVID19_Risk, PostCOVID19_DynamicRisk, by = "country_code", all.both = T)
RiskTable$Pre_Post_Difference <- RiskTable$`RiskValue_2020-05-13`- RiskTable$`RiskValue_2020-01-01`

kable(RiskTable) %>%
     kable_styling("striped", full_width = F, fixed_thead = T) %>%
     row_spec(0) %>%
     scroll_box(width = "910px", height = "440px")
```

| country\_code | RiskValue\_2020-01-01 | RiskRank\_2020-01-01 | RiskValue\_2020-05-13 | RiskRank\_2020-05-13 | Pre\_Post\_Difference |
| --- | --- | --- | --- | --- | --- |
| ABW | 2.049788 | 2 | 3.296302 | 7 | 1.2465139 |
| AFG | 1.859515 | 1 | 2.783310 | 6 | 0.9237954 |
| AGO | 1.740866 | 1 | 2.656268 | 6 | 0.9154018 |
| ALB | 2.216360 | 2 | 3.161927 | 7 | 0.9455673 |
| AND | 3.141780 | 4 | 3.314201 | 7 | 0.1724215 |
| ARE | 1.967624 | 2 | 3.082766 | 6 | 1.1151420 |
| ARG | 2.845642 | 3 | 3.348778 | 7 | 0.5031363 |
| AUS | 3.961335 | 5 | 4.256894 | 9 | 0.2955584 |
| AUT | 4.615223 | 6 | 4.578146 | 9 | -0.0370773 |
| AZE | 3.060521 | 4 | 3.423378 | 7 | 0.3628569 |
| BDI | 1.771411 | 1 | 2.612776 | 5 | 0.8413650 |
| BEL | 4.243024 | 6 | 4.634813 | 10 | 0.3917897 |
| BEN | 1.686840 | 1 | 2.517893 | 5 | 0.8310523 |
| BFA | 2.059039 | 2 | 2.682737 | 6 | 0.6236974 |
| BGD | 1.464541 | 1 | 2.821813 | 6 | 1.3572726 |
| BGR | 4.004444 | 5 | 3.959907 | 8 | -0.0445372 |
| BHR | 1.731615 | 1 | 3.203328 | 7 | 1.4717130 |
| BIH | 3.118079 | 4 | 3.573403 | 7 | 0.4553236 |
| BLZ | 1.644636 | 1 | 2.667209 | 6 | 1.0225726 |
| BMU | 3.836967 | 5 | 4.479497 | 9 | 0.6425302 |
| BOL | 1.587078 | 1 | 2.994474 | 6 | 1.4073966 |
| BRA | 2.841753 | 3 | 3.542368 | 7 | 0.7006152 |
| BRB | 3.275399 | 4 | 3.768276 | 8 | 0.4928761 |
| BRN | 2.306871 | 2 | 3.196781 | 7 | 0.8899106 |
| BWA | 1.587078 | 1 | 2.745534 | 6 | 1.1584557 |
| CAN | 3.498625 | 4 | 3.856769 | 8 | 0.3581442 |
| CHE | 4.723332 | 6 | 4.826419 | 10 | 0.1030861 |
| CHL | 2.059039 | 2 | 3.104352 | 6 | 1.0453122 |
| CHN | 2.960411 | 3 | 3.317009 | 7 | 0.3565979 |
| CIV | 1.744398 | 1 | 2.826156 | 6 | 1.0817574 |
| CMR | 1.583546 | 1 | 2.812779 | 6 | 1.2292330 |
| COD | 1.871174 | 1 | 2.851881 | 6 | 0.9807074 |
| COG | 1.744398 | 1 | 2.605516 | 5 | 0.8611171 |
| COL | 1.744398 | 1 | 2.858903 | 6 | 1.1145051 |
| CPV | 1.644636 | 1 | 2.798232 | 6 | 1.1535957 |
| CRI | 1.644636 | 1 | 3.184911 | 7 | 1.5402753 |
| CUB | 4.104207 | 5 | 4.133201 | 9 | 0.0289947 |
| CYP | 2.736628 | 3 | 3.660839 | 8 | 0.9242113 |
| CZE | 4.252275 | 6 | 4.142451 | 9 | -0.1098242 |
| DEU | 4.972068 | 7 | 4.649358 | 10 | -0.3227097 |
| DJI | 1.587078 | 1 | 2.694359 | 6 | 1.1072812 |
| DMA | 2.531001 | 3 | 3.277611 | 7 | 0.7466099 |
| DNK | 4.176214 | 5 | 4.580976 | 9 | 0.4047616 |
| DOM | 1.529520 | 1 | 2.865437 | 6 | 1.3359170 |
| DZA | 1.901719 | 2 | 2.885466 | 6 | 0.9837474 |
| ECU | 1.744398 | 1 | 3.019065 | 6 | 1.2746669 |
| EGY | 1.568355 | 1 | 2.854683 | 6 | 1.2863281 |
| ESP | 3.250794 | 4 | 3.812168 | 8 | 0.5613742 |
| EST | 3.837872 | 5 | 4.032945 | 8 | 0.1950729 |
| ETH | 1.399212 | 1 | 2.579463 | 5 | 1.1802511 |
| FIN | 4.557665 | 6 | 4.747634 | 10 | 0.1899689 |
| FRA | 3.985941 | 5 | 4.484704 | 9 | 0.4987627 |
| GAB | 2.001481 | 2 | 2.879259 | 6 | 0.8777781 |
| GBR | 3.613742 | 4 | 4.303527 | 9 | 0.6897857 |
| GHA | 1.671649 | 1 | 2.778949 | 6 | 1.1073003 |
| GMB | 1.529520 | 1 | 2.570194 | 5 | 1.0406746 |
| GRC | 3.895430 | 5 | 4.005370 | 8 | 0.1099402 |
| GRL | 3.928383 | 5 | 4.149890 | 9 | 0.2215078 |
| GTM | 1.372199 | 1 | 2.759347 | 6 | 1.3871474 |
| GUM | 2.778832 | 3 | 1.713538 | 4 | -1.0652948 |
| GUY | 1.587078 | 1 | 2.796599 | 6 | 1.2095213 |
| HND | 1.429757 | 1 | 2.633678 | 5 | 1.2039211 |
| HRV | 3.747361 | 5 | 4.100348 | 8 | 0.3529872 |
| HUN | 3.747361 | 5 | 4.003819 | 8 | 0.2564580 |
| IDN | 1.410515 | 1 | 2.698149 | 6 | 1.2876344 |
| IND | 2.031679 | 2 | 2.937126 | 6 | 0.9054468 |
| IRL | 3.836967 | 5 | 3.981641 | 8 | 0.1446736 |
| IRN | 1.587078 | 1 | 2.703460 | 6 | 1.1163818 |
| IRQ | 1.429757 | 1 | 2.596686 | 5 | 1.1669283 |
| ISL | 3.961335 | 5 | 4.306732 | 9 | 0.3453965 |
| ISR | 2.827139 | 3 | 3.707960 | 8 | 0.8808210 |
| ITA | 3.523231 | 4 | 4.257110 | 9 | 0.7338790 |
| JAM | 1.801956 | 1 | 2.853692 | 6 | 1.0517353 |
| JOR | 1.644636 | 1 | 2.829120 | 6 | 1.1844845 |
| JPN | 4.856952 | 7 | 4.792695 | 10 | -0.0642571 |
| KAZ | 3.102725 | 4 | 3.443588 | 7 | 0.3408627 |
| KEN | 1.541341 | 1 | 2.843747 | 6 | 1.3024054 |
| KGZ | 2.588559 | 3 | 3.145173 | 7 | 0.5566138 |
| KOR | 3.592448 | 4 | 3.984578 | 8 | 0.3921302 |
| KWT | 2.537103 | 3 | 3.472424 | 7 | 0.9353212 |
| LAO | 1.429757 | 1 | 2.640228 | 6 | 1.2104707 |
| LBN | 2.067329 | 2 | 3.135612 | 7 | 1.0682829 |
| LBR | 1.686840 | 1 | 2.571254 | 5 | 0.8844140 |
| LBY | 2.373681 | 2 | 3.155326 | 7 | 0.7816454 |
| LKA | 2.001481 | 2 | 3.048268 | 6 | 1.0467865 |
| LUX | 4.290426 | 6 | 4.226443 | 9 | -0.0639829 |
| MAR | 1.456770 | 1 | 2.764292 | 6 | 1.3075214 |
| MDA | 3.375162 | 4 | 3.454685 | 7 | 0.0795234 |
| MDG | 1.686840 | 1 | 2.708446 | 6 | 1.0216054 |
| MEX | 1.928732 | 2 | 3.062896 | 6 | 1.1341642 |
| MLI | 2.212828 | 2 | 2.893555 | 6 | 0.6807272 |
| MMR | 1.713853 | 1 | 2.708785 | 6 | 0.9949321 |
| MNG | 2.531001 | 3 | 3.208902 | 7 | 0.6779009 |
| MOZ | 1.786603 | 1 | 2.563266 | 5 | 0.7766632 |
| MRT | 1.429757 | 1 | 3.892820 | 8 | 2.4630626 |
| MUS | 2.531001 | 3 | 3.198590 | 7 | 0.6675891 |
| MWI | 1.744398 | 1 | 2.668853 | 6 | 0.9244542 |
| MYS | 1.686840 | 1 | 2.939672 | 6 | 1.2528317 |
| NAM | 1.959277 | 2 | 2.713307 | 6 | 0.7540300 |
| NER | 2.001481 | 2 | 2.638677 | 6 | 0.6371957 |
| NGA | 1.940034 | 2 | 2.643588 | 6 | 0.7035542 |
| NIC | 1.372199 | 1 | 2.583300 | 5 | 1.2111011 |
| NLD | 4.085703 | 5 | 4.558575 | 9 | 0.4728718 |
| NOR | 4.738686 | 6 | 5.103268 | 10 | 0.3645817 |
| NZL | 3.556183 | 4 | 4.053504 | 8 | 0.4973205 |
| OMN | 1.801956 | 1 | 2.956411 | 6 | 1.1544549 |
| PAK | 1.967047 | 2 | 2.780630 | 6 | 0.8135830 |
| PAN | 1.801956 | 1 | 2.777455 | 6 | 0.9754980 |
| PER | 1.587078 | 1 | 3.027605 | 6 | 1.4405268 |
| PHL | 1.713853 | 1 | 2.710774 | 6 | 0.9969212 |
| PNG | 2.216360 | 2 | 2.683891 | 6 | 0.4675313 |
| POL | 3.432720 | 4 | 3.835263 | 8 | 0.4025433 |
| PRI | 2.993711 | 3 | 1.860040 | 4 | -1.1336709 |
| PRT | 3.680551 | 5 | 4.085837 | 8 | 0.4052854 |
| PRY | 1.529520 | 1 | 2.859919 | 6 | 1.3303989 |
| PSE | 1.314641 | 1 | 2.566045 | 5 | 1.2514036 |
| QAT | 1.943181 | 2 | 3.545923 | 7 | 1.6027417 |
| ROU | 3.532482 | 4 | 3.887895 | 8 | 0.3554121 |
| RUS | 4.200080 | 5 | 4.008191 | 8 | -0.1918888 |
| RWA | 1.456770 | 1 | 2.599422 | 5 | 1.1426521 |
| SAU | 2.049788 | 2 | 3.040989 | 6 | 0.9912008 |
| SDN | 1.372199 | 1 | 2.772478 | 6 | 1.4002786 |
| SEN | 1.456770 | 1 | 2.698837 | 6 | 1.2420670 |
| SGP | 2.771988 | 3 | 3.765718 | 8 | 0.9937296 |
| SLE | 2.028494 | 2 | 3.375990 | 7 | 1.3474951 |
| SLV | 1.901719 | 2 | 2.878916 | 6 | 0.9771966 |
| SMR | 3.928383 | 5 | 4.992411 | 10 | 1.0640288 |
| SOM | 1.786603 | 1 | 2.595345 | 5 | 0.8087423 |
| SRB | 3.590041 | 4 | 3.808916 | 8 | 0.2188753 |
| SSD | 2.158802 | 2 | 2.701773 | 6 | 0.5429707 |
| SUR | 2.001481 | 2 | 3.036616 | 6 | 1.0351348 |
| SVK | 3.665198 | 5 | 3.822979 | 8 | 0.1577812 |
| SVN | 3.507877 | 4 | 4.061166 | 8 | 0.5532887 |
| SWE | 4.070349 | 5 | 4.561716 | 9 | 0.4913666 |
| SWZ | 1.844161 | 1 | 2.811018 | 6 | 0.9668574 |
| SYC | 2.001481 | 2 | 2.995922 | 6 | 0.9944403 |
| SYR | 1.686840 | 1 | 2.663486 | 6 | 0.9766461 |
| TCD | 2.158802 | 2 | 2.705569 | 6 | 0.5467671 |
| THA | 2.086053 | 2 | 3.095848 | 6 | 1.0097956 |
| TTO | 2.803438 | 3 | 3.343645 | 7 | 0.5402070 |
| TUN | 2.174156 | 2 | 2.975786 | 6 | 0.8016298 |
| TUR | 2.273918 | 2 | 3.006302 | 6 | 0.7323838 |
| TZA | 1.556533 | 1 | 2.574917 | 5 | 1.0183843 |
| UGA | 1.556533 | 1 | 2.579931 | 5 | 1.0233979 |
| UKR | 4.004444 | 5 | 3.932455 | 8 | -0.0719892 |
| URY | 3.118079 | 4 | 3.803544 | 8 | 0.6854653 |
| USA | 3.665767 | 5 | 4.177113 | 9 | 0.5113462 |
| UZB | 2.872655 | 3 | 3.509301 | 7 | 0.6366458 |
| VEN | 1.744398 | 1 | 2.927415 | 6 | 1.1830165 |
| VNM | 1.798425 | 1 | 2.894156 | 6 | 1.0957320 |
| YEM | 1.556533 | 1 | 2.959244 | 6 | 1.4027115 |
| ZAF | 2.331476 | 2 | 3.061768 | 6 | 0.7302918 |
| ZMB | 1.901719 | 2 | 2.641078 | 6 | 0.7393593 |
| ZWE | 1.801956 | 1 | 2.658108 | 6 | 0.8561513 |

```
sp_data <- RiskTable[, c(1, 6)]
map <- worldPlot(sp_data)
map
```

```
## Scale on map varies by more than 10%, scale bar may be inaccurate
```

# 7 List of Post-COVID-19 Top Countries with Level of Risk

```
data_Covid <- covid_data_day(SI.date, df_c, df_d, df_r)
```

```
## `summarise()` ungrouping output (override with `.groups` argument)
## `summarise()` ungrouping output (override with `.groups` argument)
## `summarise()` ungrouping output (override with `.groups` argument)
```

```
countryList <- CountryCodeList
colnames(countryList) <- c("country", "country_code")
data_Covid <- merge(data_Covid, countryList, by = 'country', all.x = T)

TopCountries <- merge(data_Covid, RiskTable, by = "country_code", all.both=T)

#----- Data Integration
TopCountries_Base <- merge(TopCountries, BaseData, by = "country_code", all.x = T)

TopCountries_Base <- TopCountries_Base[,c(1:11,13)]
TopCountries_Base$MortalityRate <- round(TopCountries_Base$deaths / TopCountries_Base$Pop_2018 * 1000000,1)

TopCountries_Base <- TopCountries_Base[order(-TopCountries_Base[13]),]


kable(TopCountries_Base) %>%
     kable_styling("striped", full_width = F, fixed_thead = T) %>%
     row_spec(0) %>%
     scroll_box(width = "910px", height = "440px")
```

|  | country\_code | country | confirmed | recovered | deaths | active | RiskValue\_2020-01-01 | RiskRank\_2020-01-01 | RiskValue\_2020-05-13 | RiskRank\_2020-05-13 | Pre\_Post\_Difference | Pop\_2018 | MortalityRate |
| --- | --- | --- | --- | --- | --- | --- | --- | --- | --- | --- | --- | --- | --- |
| 125 | SMR | San Marino | 643 | 161 | 41 | 482 | 3.928383 | 5 | 4.992411 | 10 | 1.0640288 | 33785 | 1213.6 |
| 12 | BEL | Belgium | 53981 | 13937 | 8843 | 40044 | 4.243024 | 6 | 4.634813 | 10 | 0.3917897 | 11422068 | 774.2 |
| 5 | AND | Andorra | 760 | 576 | 49 | 184 | 3.141780 | 4 | 3.314201 | 7 | 0.1724215 | 77006 | 636.3 |
| 48 | ESP | Spain | 228691 | 140823 | 27104 | 87868 | 3.250794 | 4 | 3.812168 | 8 | 0.5613742 | 46723749 | 580.1 |
| 71 | ITA | Italy | 222104 | 112541 | 31106 | 109563 | 3.523231 | 4 | 4.257110 | 9 | 0.7338790 | 60431283 | 514.7 |
| 54 | GBR | United Kingdom | 229705 | 0 | 33186 | 229705 | 3.613742 | 4 | 4.303527 | 9 | 0.6897857 | 66488991 | 499.1 |
| 52 | FRA | France | 175981 | 57368 | 27032 | 118613 | 3.985941 | 5 | 4.484704 | 9 | 0.4987627 | 66987244 | 403.5 |
| 132 | SWE | Sweden | 27909 | 4971 | 3460 | 22938 | 4.070349 | 5 | 4.561716 | 9 | 0.4913666 | 10183175 | 339.8 |
| 102 | NLD | Netherlands | 43217 | 6 | 5562 | 43211 | 4.085703 | 5 | 4.558575 | 9 | 0.4728718 | 17231017 | 322.8 |
| 66 | IRL | Ireland | 23401 | 19470 | 1497 | 3931 | 3.836967 | 5 | 3.981641 | 8 | 0.1446736 | 4853506 | 308.4 |
| 145 | USA | US | 1390406 | 243430 | 84119 | 1146976 | 3.665767 | 5 | 4.177113 | 9 | 0.5113462 | 327167434 | 257.1 |
| 27 | CHE | Switzerland | 30413 | 27100 | 1870 | 3313 | 4.723332 | 6 | 4.826419 | 10 | 0.1030861 | 8516543 | 219.6 |
| 85 | LUX | Luxembourg | 3904 | 3629 | 103 | 275 | 4.290426 | 6 | 4.226443 | 9 | -0.0639829 | 607728 | 169.5 |
| 26 | CAN | Canada | 73568 | 35177 | 5425 | 38391 | 3.498625 | 4 | 3.856769 | 8 | 0.3581442 | 37058856 | 146.4 |
| 46 | ECU | Ecuador | 30486 | 3433 | 2334 | 27053 | 1.744398 | 1 | 3.019065 | 6 | 1.2746669 | 17084357 | 136.6 |
| 20 | BMU | Bermuda | 121 | 66 | 8 | 55 | 3.836967 | 5 | 4.479497 | 9 | 0.6425302 | 63968 | 125.1 |
| 112 | PRT | Portugal | 28132 | 3182 | 1175 | 24950 | 3.680551 | 5 | 4.085837 | 8 | 0.4052854 | 10281762 | 114.3 |
| 40 | DEU | Germany | 174098 | 148700 | 7861 | 25398 | 4.972068 | 7 | 4.649358 | 10 | -0.3227097 | 82927922 | 94.8 |
| 43 | DNK | Denmark | 10667 | 8663 | 533 | 2004 | 4.176214 | 5 | 4.580976 | 9 | 0.4047616 | 5797446 | 91.9 |
| 67 | IRN | Iran | 112725 | 89428 | 6783 | 23297 | 1.587078 | 1 | 2.703460 | 6 | 1.1163818 | 81800269 | 82.9 |
| 9 | AUT | Austria | 15997 | 14304 | 624 | 1693 | 4.615223 | 6 | 4.578146 | 9 | -0.0370773 | 8847037 | 70.5 |
| 108 | PER | Peru | 76306 | 24324 | 2169 | 51982 | 1.587078 | 1 | 3.027605 | 6 | 1.4405268 | 31989256 | 67.8 |
| 22 | BRA | Brazil | 190137 | 78424 | 13240 | 111713 | 2.841753 | 3 | 3.542368 | 7 | 0.7006152 | 209469333 | 63.2 |
| 107 | PAN | Panama | 8944 | 6067 | 256 | 2877 | 1.801956 | 1 | 2.777455 | 6 | 0.9754980 | 4176873 | 61.3 |
| 116 | ROU | Romania | 16002 | 7961 | 1036 | 8041 | 3.532482 | 4 | 3.887895 | 8 | 0.3554121 | 19473936 | 53.2 |
| 87 | MDA | Moldova | 5406 | 2176 | 185 | 3230 | 3.375162 | 4 | 3.454685 | 7 | 0.0795234 | 3545883 | 52.2 |
| 51 | FIN | Finland | 6054 | 4300 | 284 | 1754 | 4.557665 | 6 | 4.747634 | 10 | 0.1899689 | 5518050 | 51.5 |
| 131 | SVN | Slovenia | 1463 | 260 | 103 | 1203 | 3.507877 | 4 | 4.061166 | 8 | 0.5532887 | 2067372 | 49.8 |
| 140 | TUR | Turkey | 143114 | 101715 | 3952 | 41399 | 2.273918 | 2 | 3.006302 | 6 | 0.7323838 | 82319724 | 48.0 |
| 49 | EST | Estonia | 1751 | 777 | 61 | 974 | 3.837872 | 5 | 4.032945 | 8 | 0.1950729 | 1320884 | 46.2 |
| 63 | HUN | Hungary | 3341 | 1102 | 430 | 2239 | 3.747361 | 5 | 4.003819 | 8 | 0.2564580 | 9768785 | 44.0 |
| 103 | NOR | Norway | 8175 | 32 | 229 | 8143 | 4.738686 | 6 | 5.103268 | 10 | 0.3645817 | 5314336 | 43.1 |
| 44 | DOM | Dominican Republic | 11196 | 3221 | 409 | 7975 | 1.529520 | 1 | 2.865437 | 6 | 1.3359170 | 10627165 | 38.5 |
| 18 | BIH | Bosnia and Herzegovina | 2181 | 1228 | 120 | 953 | 3.118079 | 4 | 3.573403 | 7 | 0.4553236 | 3323929 | 36.1 |
| 89 | MEX | Mexico | 40186 | 26990 | 4220 | 13196 | 1.928732 | 2 | 3.062896 | 6 | 1.1341642 | 126190788 | 33.4 |
| 127 | SRB | Serbia | 10295 | 3824 | 222 | 6471 | 3.590041 | 4 | 3.808916 | 8 | 0.2188753 | 6982084 | 31.8 |
| 70 | ISR | Israel | 16548 | 12232 | 264 | 4316 | 2.827139 | 3 | 3.707960 | 8 | 0.8808210 | 8883800 | 29.7 |
| 1 | ABW | Aruba | 101 | 91 | 3 | 10 | 2.049788 | 2 | 3.296302 | 7 | 1.2465139 | 105845 | 28.3 |
| 69 | ISL | Iceland | 1802 | 1780 | 10 | 22 | 3.961335 | 5 | 4.306732 | 9 | 0.3453965 | 353574 | 28.3 |
| 39 | CZE | Czechia | 8269 | 5047 | 290 | 3222 | 4.252275 | 6 | 4.142451 | 9 | -0.1098242 | 10625695 | 27.3 |
| 23 | BRB | Barbados | 85 | 65 | 7 | 20 | 3.275399 | 4 | 3.768276 | 8 | 0.4928761 | 286641 | 24.4 |
| 62 | HRV | Croatia | 2213 | 1834 | 94 | 379 | 3.747361 | 5 | 4.100348 | 8 | 0.3529872 | 4089400 | 23.0 |
| 111 | POL | Poland | 17204 | 6410 | 861 | 10794 | 3.432720 | 4 | 3.835263 | 8 | 0.4025433 | 37978548 | 22.7 |
| 6 | ARE | United Arab Emirates | 20386 | 6523 | 206 | 13863 | 1.967624 | 2 | 3.082766 | 6 | 1.1151420 | 9630959 | 21.4 |
| 79 | KWT | Kuwait | 11028 | 3263 | 82 | 7765 | 2.537103 | 3 | 3.472424 | 7 | 0.9353212 | 4137309 | 19.8 |
| 28 | CHL | Chile | 34381 | 14865 | 346 | 19516 | 2.059039 | 2 | 3.104352 | 6 | 1.0453122 | 18729160 | 18.5 |
| 117 | RUS | Russia | 242271 | 48003 | 2212 | 194268 | 4.200080 | 5 | 4.008191 | 8 | -0.1918888 | 144478050 | 15.3 |
| 57 | GRC | Greece | 2760 | 1374 | 155 | 1386 | 3.895430 | 5 | 4.005370 | 8 | 0.1099402 | 10727668 | 14.4 |
| 38 | CYP | Cyprus | 905 | 449 | 17 | 456 | 2.736628 | 3 | 3.660839 | 8 | 0.9242113 | 1189265 | 14.3 |
| 16 | BGR | Bulgaria | 2069 | 499 | 96 | 1570 | 4.004444 | 5 | 3.959907 | 8 | -0.0445372 | 7024216 | 13.7 |
| 60 | GUY | Guyana | 113 | 41 | 10 | 72 | 1.587078 | 1 | 2.796599 | 6 | 1.2095213 | 779004 | 12.8 |
| 61 | HND | Honduras | 2255 | 237 | 123 | 2018 | 1.429757 | 1 | 2.633678 | 5 | 1.2039211 | 9587522 | 12.8 |
| 21 | BOL | Bolivia | 3148 | 339 | 142 | 2809 | 1.587078 | 1 | 2.994474 | 6 | 1.4073966 | 11353142 | 12.5 |
| 45 | DZA | Algeria | 6253 | 3058 | 522 | 3195 | 1.901719 | 2 | 2.885466 | 6 | 0.9837474 | 42228429 | 12.4 |
| 4 | ALB | Albania | 880 | 688 | 31 | 192 | 2.216360 | 2 | 3.161927 | 7 | 0.9455673 | 2866376 | 10.8 |
| 34 | COL | Colombia | 12930 | 3133 | 509 | 9797 | 1.744398 | 1 | 2.858903 | 6 | 1.1145051 | 49648685 | 10.3 |
| 143 | UKR | Ukraine | 16425 | 3716 | 439 | 12709 | 4.004444 | 5 | 3.932455 | 8 | -0.0719892 | 44622516 | 9.8 |
| 119 | SAU | Saudi Arabia | 44830 | 17622 | 273 | 27208 | 2.049788 | 2 | 3.040989 | 6 | 0.9912008 | 33699947 | 8.1 |
| 95 | MUS | Mauritius | 332 | 322 | 10 | 10 | 2.531001 | 3 | 3.198590 | 7 | 0.6675891 | 1265303 | 7.9 |
| 7 | ARG | Argentina | 6879 | 2266 | 329 | 4613 | 2.845642 | 3 | 3.348778 | 7 | 0.5031363 | 44494502 | 7.4 |
| 109 | PHL | Philippines | 11618 | 2251 | 772 | 9367 | 1.713853 | 1 | 2.710774 | 6 | 0.9969212 | 106651922 | 7.2 |
| 37 | CUB | Cuba | 1810 | 1326 | 79 | 484 | 4.104207 | 5 | 4.133201 | 9 | 0.0289947 | 11338138 | 7.0 |
| 17 | BHR | Bahrain | 5816 | 2205 | 10 | 3611 | 1.731615 | 1 | 3.203328 | 7 | 1.4717130 | 1569439 | 6.4 |
| 138 | TTO | Trinidad and Tobago | 116 | 107 | 8 | 9 | 2.803438 | 3 | 3.343645 | 7 | 0.5402070 | 1389858 | 5.8 |
| 47 | EGY | Egypt | 10431 | 2486 | 556 | 7945 | 1.568355 | 1 | 2.854683 | 6 | 1.2863281 | 98423595 | 5.6 |
| 144 | URY | Uruguay | 719 | 545 | 19 | 174 | 3.118079 | 4 | 3.803544 | 8 | 0.6854653 | 3449299 | 5.5 |
| 31 | CMR | Cameroon | 2800 | 1543 | 136 | 1257 | 1.583546 | 1 | 2.812779 | 6 | 1.2292330 | 25216237 | 5.4 |
| 74 | JPN | Japan | 16049 | 8920 | 678 | 7129 | 4.856952 | 7 | 4.792695 | 10 | -0.0642571 | 126529100 | 5.4 |
| 19 | BLZ | Belize | 18 | 16 | 2 | 2 | 1.644636 | 1 | 2.667209 | 6 | 1.0225726 | 383071 | 5.2 |
| 86 | MAR | Morocco | 6512 | 3131 | 188 | 3381 | 1.456770 | 1 | 2.764292 | 6 | 1.3075214 | 36029138 | 5.2 |
| 78 | KOR | Korea, South | 10991 | 9762 | 260 | 1229 | 3.592448 | 4 | 3.984578 | 8 | 0.3921302 | 51635256 | 5.0 |
| 115 | QAT | Qatar | 26539 | 3143 | 14 | 23396 | 1.943181 | 2 | 3.545923 | 7 | 1.6027417 | 2781677 | 5.0 |
| 130 | SVK | Slovakia | 1469 | 1060 | 27 | 409 | 3.665198 | 5 | 3.822979 | 8 | 0.1577812 | 5447011 | 5.0 |
| 104 | NZL | New Zealand | 1497 | 1411 | 21 | 86 | 3.556183 | 4 | 4.053504 | 8 | 0.4973205 | 4885500 | 4.3 |
| 53 | GAB | Gabon | 1004 | 152 | 9 | 852 | 2.001481 | 2 | 2.879259 | 6 | 0.8777781 | 2119275 | 4.2 |
| 82 | LBR | Liberia | 213 | 101 | 20 | 112 | 1.686840 | 1 | 2.571254 | 5 | 0.8844140 | 4818977 | 4.2 |
| 8 | AUS | Australia | 6989 | 6297 | 98 | 692 | 3.961335 | 5 | 4.256894 | 9 | 0.2955584 | 24992369 | 3.9 |
| 139 | TUN | Tunisia | 1032 | 759 | 45 | 273 | 2.174156 | 2 | 2.975786 | 6 | 0.8016298 | 11565204 | 3.9 |
| 64 | IDN | Indonesia | 15438 | 3287 | 1028 | 12151 | 1.410515 | 1 | 2.698149 | 6 | 1.2876344 | 267663435 | 3.8 |
| 81 | LBN | Lebanon | 878 | 236 | 26 | 642 | 2.067329 | 2 | 3.135612 | 7 | 1.0682829 | 6848925 | 3.8 |
| 150 | ZAF | South Africa | 12074 | 4745 | 219 | 7329 | 2.331476 | 2 | 3.061768 | 6 | 0.7302918 | 57779622 | 3.8 |
| 35 | CPV | Cabo Verde | 289 | 61 | 2 | 228 | 1.644636 | 1 | 2.798232 | 6 | 1.1535957 | 543767 | 3.7 |
| 122 | SGP | Singapore | 25346 | 4809 | 21 | 20537 | 2.771988 | 3 | 3.765718 | 8 | 0.9937296 | 5638676 | 3.7 |
| 2 | AFG | Afghanistan | 5226 | 648 | 132 | 4578 | 1.859515 | 1 | 2.783310 | 6 | 0.9237954 | 37172386 | 3.6 |
| 106 | PAK | Pakistan | 35298 | 8899 | 761 | 26399 | 1.967047 | 2 | 2.780630 | 6 | 0.8135830 | 212215030 | 3.6 |
| 10 | AZE | Azerbaijan | 2758 | 1789 | 35 | 969 | 3.060521 | 4 | 3.423378 | 7 | 0.3628569 | 9942334 | 3.5 |
| 97 | MYS | Malaysia | 6779 | 5281 | 111 | 1498 | 1.686840 | 1 | 2.939672 | 6 | 1.2528317 | 31528585 | 3.5 |
| 105 | OMN | Oman | 4019 | 1289 | 17 | 2730 | 1.801956 | 1 | 2.956411 | 6 | 1.1544549 | 4829483 | 3.5 |
| 126 | SOM | Somalia | 1219 | 130 | 52 | 1089 | 1.786603 | 1 | 2.595345 | 5 | 0.8087423 | 15008154 | 3.5 |
| 123 | SLE | Sierra Leone | 387 | 97 | 26 | 290 | 2.028494 | 2 | 3.375990 | 7 | 1.3474951 | 7650154 | 3.4 |
| 29 | CHN | China | 82974 | 78238 | 4633 | 4736 | 2.960411 | 3 | 3.317009 | 7 | 0.3565979 | 1392730000 | 3.3 |
| 41 | DJI | Djibouti | 1268 | 900 | 3 | 368 | 1.587078 | 1 | 2.694359 | 6 | 1.1072812 | 958920 | 3.1 |
| 72 | JAM | Jamaica | 509 | 113 | 9 | 396 | 1.801956 | 1 | 2.853692 | 6 | 1.0517353 | 2934855 | 3.1 |
| 124 | SLV | El Salvador | 1037 | 374 | 20 | 663 | 1.901719 | 2 | 2.878916 | 6 | 0.9771966 | 6420744 | 3.1 |
| 68 | IRQ | Iraq | 3032 | 1966 | 115 | 1066 | 1.429757 | 1 | 2.596686 | 5 | 1.1669283 | 38433600 | 3.0 |
| 136 | TCD | Chad | 372 | 78 | 42 | 294 | 2.158802 | 2 | 2.705569 | 6 | 0.5467671 | 15477751 | 2.7 |
| 14 | BFA | Burkina Faso | 773 | 592 | 51 | 181 | 2.059039 | 2 | 2.682737 | 6 | 0.6236974 | 19751535 | 2.6 |
| 24 | BRN | Brunei | 141 | 134 | 1 | 7 | 2.306871 | 2 | 3.196781 | 7 | 0.8899106 | 428962 | 2.3 |
| 90 | MLI | Mali | 758 | 412 | 44 | 346 | 2.212828 | 2 | 2.893555 | 6 | 0.6807272 | 19077690 | 2.3 |
| 99 | NER | Niger | 860 | 658 | 49 | 202 | 2.001481 | 2 | 2.638677 | 6 | 0.6371957 | 22442948 | 2.2 |
| 120 | SDN | Sudan | 1818 | 198 | 90 | 1620 | 1.372199 | 1 | 2.772478 | 6 | 1.4002786 | 41801533 | 2.2 |
| 33 | COG | Congo (Brazzaville) | 333 | 53 | 11 | 280 | 1.744398 | 1 | 2.605516 | 5 | 0.8611171 | 5244363 | 2.1 |
| 65 | IND | India | 78055 | 26400 | 2551 | 51655 | 2.031679 | 2 | 2.937126 | 6 | 0.9054468 | 1352617328 | 1.9 |
| 77 | KGZ | Kyrgyzstan | 1044 | 726 | 12 | 318 | 2.588559 | 3 | 3.145173 | 7 | 0.5566138 | 6315800 | 1.9 |
| 75 | KAZ | Kazakhstan | 5417 | 2408 | 32 | 3009 | 3.102725 | 4 | 3.443588 | 7 | 0.3408627 | 18276499 | 1.8 |
| 133 | SWZ | Eswatini | 187 | 48 | 2 | 139 | 1.844161 | 1 | 2.811018 | 6 | 0.9668574 | 1136191 | 1.8 |
| 15 | BGD | Bangladesh | 17822 | 3361 | 269 | 14461 | 1.464541 | 1 | 2.821813 | 6 | 1.3572726 | 161356039 | 1.7 |
| 59 | GTM | Guatemala | 1342 | 121 | 29 | 1221 | 1.372199 | 1 | 2.759347 | 6 | 1.3871474 | 17247807 | 1.7 |
| 129 | SUR | Suriname | 10 | 9 | 1 | 1 | 2.001481 | 2 | 3.036616 | 6 | 1.0351348 | 575991 | 1.7 |
| 36 | CRI | Costa Rica | 815 | 527 | 8 | 288 | 1.644636 | 1 | 3.184911 | 7 | 1.5402753 | 4999441 | 1.6 |
| 113 | PRY | Paraguay | 740 | 182 | 11 | 558 | 1.529520 | 1 | 2.859919 | 6 | 1.3303989 | 6956071 | 1.6 |
| 121 | SEN | Senegal | 2105 | 782 | 21 | 1323 | 1.456770 | 1 | 2.698837 | 6 | 1.2420670 | 15854360 | 1.3 |
| 101 | NIC | Nicaragua | 25 | 7 | 8 | 18 | 1.372199 | 1 | 2.583300 | 5 | 1.2111011 | 6465513 | 1.2 |
| 30 | CIV | Cote d’Ivoire | 1912 | 902 | 24 | 1010 | 1.744398 | 1 | 2.826156 | 6 | 1.0817574 | 25069229 | 1.0 |
| 73 | JOR | Jordan | 582 | 392 | 9 | 190 | 1.644636 | 1 | 2.829120 | 6 | 1.1844845 | 9956011 | 0.9 |
| 55 | GHA | Ghana | 5408 | 514 | 24 | 4894 | 1.671649 | 1 | 2.778949 | 6 | 1.1073003 | 29767108 | 0.8 |
| 76 | KEN | Kenya | 737 | 281 | 40 | 456 | 1.541341 | 1 | 2.843747 | 6 | 1.3024054 | 51393010 | 0.8 |
| 100 | NGA | Nigeria | 4971 | 1070 | 164 | 3901 | 1.940034 | 2 | 2.643588 | 6 | 0.7035542 | 195874740 | 0.8 |
| 137 | THA | Thailand | 3017 | 2844 | 56 | 173 | 2.086053 | 2 | 3.095848 | 6 | 1.0097956 | 69428524 | 0.8 |
| 32 | COD | Congo (Kinshasa) | 1169 | 148 | 50 | 1021 | 1.871174 | 1 | 2.851881 | 6 | 0.9807074 | 84068091 | 0.6 |
| 94 | MRT | Mauritania | 15 | 6 | 2 | 9 | 1.429757 | 1 | 3.892820 | 8 | 2.4630626 | 4403319 | 0.5 |
| 25 | BWA | Botswana | 24 | 17 | 1 | 7 | 1.587078 | 1 | 2.745534 | 6 | 1.1584557 | 2254126 | 0.4 |
| 56 | GMB | Gambia | 23 | 10 | 1 | 13 | 1.529520 | 1 | 2.570194 | 5 | 1.0406746 | 2280102 | 0.4 |
| 83 | LBY | Libya | 64 | 28 | 3 | 36 | 2.373681 | 2 | 3.155326 | 7 | 0.7816454 | 6678567 | 0.4 |
| 84 | LKA | Sri Lanka | 915 | 382 | 9 | 533 | 2.001481 | 2 | 3.048268 | 6 | 1.0467865 | 21670000 | 0.4 |
| 114 | PSE | West Bank and Gaza | 375 | 310 | 2 | 65 | 1.314641 | 1 | 2.566045 | 5 | 1.2514036 | 4569087 | 0.4 |
| 141 | TZA | Tanzania | 509 | 183 | 21 | 326 | 1.556533 | 1 | 2.574917 | 5 | 1.0183843 | 56318348 | 0.4 |
| 149 | YEM | Yemen | 70 | 1 | 12 | 69 | 1.556533 | 1 | 2.959244 | 6 | 1.4027115 | 28498687 | 0.4 |
| 151 | ZMB | Zambia | 446 | 124 | 7 | 322 | 1.901719 | 2 | 2.641078 | 6 | 0.7393593 | 17351822 | 0.4 |
| 146 | UZB | Uzbekistan | 2612 | 2076 | 11 | 536 | 2.872655 | 3 | 3.509301 | 7 | 0.6366458 | 32955400 | 0.3 |
| 147 | VEN | Venezuela | 423 | 220 | 10 | 203 | 1.744398 | 1 | 2.927415 | 6 | 1.1830165 | 28870195 | 0.3 |
| 152 | ZWE | Zimbabwe | 37 | 12 | 4 | 25 | 1.801956 | 1 | 2.658108 | 6 | 0.8561513 | 14439018 | 0.3 |
| 13 | BEN | Benin | 327 | 83 | 2 | 244 | 1.686840 | 1 | 2.517893 | 5 | 0.8310523 | 11485048 | 0.2 |
| 96 | MWI | Malawi | 63 | 24 | 3 | 39 | 1.744398 | 1 | 2.668853 | 6 | 0.9244542 | 18143315 | 0.2 |
| 135 | SYR | Syria | 48 | 29 | 3 | 19 | 1.686840 | 1 | 2.663486 | 6 | 0.9766461 | 16906283 | 0.2 |
| 3 | AGO | Angola | 45 | 14 | 2 | 31 | 1.740866 | 1 | 2.656268 | 6 | 0.9154018 | 30809762 | 0.1 |
| 11 | BDI | Burundi | 15 | 7 | 1 | 8 | 1.771411 | 1 | 2.612776 | 5 | 0.8413650 | 11175378 | 0.1 |
| 91 | MMR | Burma | 181 | 79 | 6 | 102 | 1.713853 | 1 | 2.708785 | 6 | 0.9949321 | 53708395 | 0.1 |
| 42 | DMA | Dominica | 16 | 15 | 0 | 1 | 2.531001 | 3 | 3.277611 | 7 | 0.7466099 | 71625 | 0.0 |
| 50 | ETH | Ethiopia | 263 | 108 | 5 | 155 | 1.399212 | 1 | 2.579463 | 5 | 1.1802511 | 109224559 | 0.0 |
| 58 | GRL | Greenland | 11 | 11 | 0 | 0 | 3.928383 | 5 | 4.149890 | 9 | 0.2215078 | 56025 | 0.0 |
| 80 | LAO | Laos | 19 | 14 | 0 | 5 | 1.429757 | 1 | 2.640228 | 6 | 1.2104707 | 7061507 | 0.0 |
| 88 | MDG | Madagascar | 212 | 107 | 0 | 105 | 1.686840 | 1 | 2.708446 | 6 | 1.0216054 | 26262368 | 0.0 |
| 92 | MNG | Mongolia | 42 | 15 | 0 | 27 | 2.531001 | 3 | 3.208902 | 7 | 0.6779009 | 3170208 | 0.0 |
| 93 | MOZ | Mozambique | 104 | 34 | 0 | 70 | 1.786603 | 1 | 2.563266 | 5 | 0.7766632 | 29495962 | 0.0 |
| 98 | NAM | Namibia | 16 | 11 | 0 | 5 | 1.959277 | 2 | 2.713307 | 6 | 0.7540300 | 2448255 | 0.0 |
| 110 | PNG | Papua New Guinea | 8 | 8 | 0 | 0 | 2.216360 | 2 | 2.683891 | 6 | 0.4675313 | 8606316 | 0.0 |
| 118 | RWA | Rwanda | 287 | 164 | 0 | 123 | 1.456770 | 1 | 2.599422 | 5 | 1.1426521 | 12301939 | 0.0 |
| 128 | SSD | South Sudan | 203 | 2 | 0 | 201 | 2.158802 | 2 | 2.701773 | 6 | 0.5429707 | 10975920 | 0.0 |
| 134 | SYC | Seychelles | 11 | 10 | 0 | 1 | 2.001481 | 2 | 2.995922 | 6 | 0.9944403 | 96762 | 0.0 |
| 142 | UGA | Uganda | 139 | 55 | 0 | 84 | 1.556533 | 1 | 2.579931 | 5 | 1.0233979 | 42723139 | 0.0 |
| 148 | VNM | Vietnam | 288 | 252 | 0 | 36 | 1.798425 | 1 | 2.894156 | 6 | 1.0957320 | 95540395 | 0.0 |
